# Cardiac contraction and relaxation are regulated by distinct subcellular cAMP pools

**DOI:** 10.1038/s41589-023-01381-8

**Published:** 2023-07-20

**Authors:** Ting-Yu Lin, Quynh N. Mai, Hao Zhang, Emily Wilson, Huan-Chieh Chien, Sook Wah Yee, Kathleen M. Giacomini, Jeffrey E. Olgin, Roshanak Irannejad

**Affiliations:** 1 Cardiovascular Research Institute, University of California, San Francisco, CA USA; 2 Department of Medicine, Division of Cardiology, University of California San Francisco, San Francisco, CA USA; 3 Department of Bioengineering and Therapeutic Sciences, University of California San Francisco, California, CA USA; 4 Institute for Human Genetics, University of California San Francisco, San Francisco, CA USA; 5 Department of Biochemistry & Biophysics, University of California, San Francisco, CA USA

**Keywords:** Cell signalling, Cardiovascular diseases, G protein-coupled receptors, Chemical tools, Model vertebrates

## Abstract

Cells interpret a variety of signals through G-protein-coupled receptors (GPCRs) and stimulate the generation of second messengers such as cyclic adenosine monophosphate (cAMP). A long-standing puzzle is deciphering how GPCRs elicit different physiological responses despite generating similar levels of cAMP. We previously showed that some GPCRs generate cAMP from both the plasma membrane and the Golgi apparatus. Here we demonstrate that cardiomyocytes distinguish between subcellular cAMP inputs to elicit different physiological outputs. We show that generating cAMP from the Golgi leads to the regulation of a specific protein kinase A (PKA) target that increases the rate of cardiomyocyte relaxation. In contrast, cAMP generation from the plasma membrane activates a different PKA target that increases contractile force. We further validated the physiological consequences of these observations in intact zebrafish and mice. Thus, we demonstrate that the same GPCR acting through the same second messenger regulates cardiac contraction and relaxation dependent on its subcellular location.

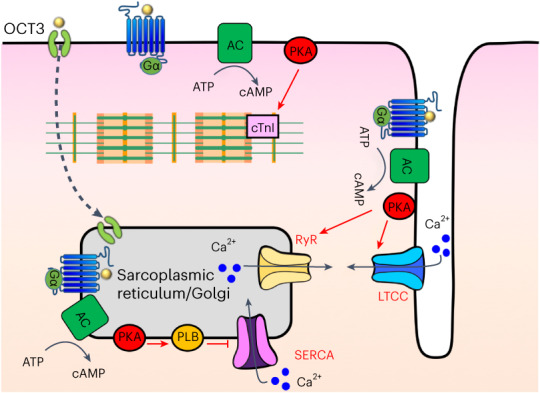

## Abstract (web summary)

A pivotal role for beta-1 adrenergic receptor (β1AR) subcellular signaling in controlling cardiac relaxation response through the generation of cyclic adenosine monophosphate (cAMP) and activation of local protein kinase A (PKA) effectors was revealed in cardiomyocytes and in intact zebrafish and mice hearts.

## Main

G-protein-coupled receptors (GPCRs) are the largest family of membrane receptors that communicate downstream signaling pathways to regulate cellular functions. Many of them signal through the stimulatory G protein (Gs) to generate cyclic adenosine monophosphate (cAMP)^1^. There are a number of different hormones that stimulate cAMP generation through the activation of different types of GPCRs that are expressed in the same cell^2^. However, activation of each GPCR can trigger different physiological responses despite generating the same level of cAMP. A classic example of such distinct physiological responses is the activation of beta-adrenergic receptors (βARs) and prostaglandin E1-type receptors, which trigger similar elevations of cAMP in cardiac tissue, but only the activated βAR causes increased cardiac contractility and glycogen metabolism in cardiomyocytes^3,4^. Analogous observations have been reported even within the same family of receptors. For example, β1AR and β2AR, the two main beta-adrenergic family isoforms in cardiomyocytes are both activated by sympathetic hormones epinephrine and norepinephrine and trigger Gs-mediated cAMP generation^5,6^. Nevertheless, these receptors elicit distinct effects on cardiac function. In healthy cardiomyocytes, β1AR signaling regulates chronotropy (heart rate), inotropy (force of contraction) and lusitropy (relaxation)^7,8^ while β2AR signaling only modestly contributes to chronotropy and has no appreciable effect on lusitropy in mice^7,8^. In the context of heart failure, β1AR signaling promotes cardiomyocyte hypertrophy and apoptosis, whereas β2AR signaling inhibits both^9–12^. Numerous hypotheses have been advanced to explain how β1AR and β2AR function differently from each other, both in health and disease states, but answers have been elusive. Notably, β1AR and β2AR localize to different subdomains in cardiomyocytes. While β1AR is mostly at the plasma and Golgi membranes, β2AR is mostly localized in transverse tubules (t-tubules). Whether the distinct βAR localizations underlie the noted physiological and pathophysiological outcomes attributed to each has not been addressed. In the past decade, several reports have shown that GPCR can signal from subcellular membrane compartments^13–20^. For example, we have shown that β1AR can be activated and generate cAMP from the plasma membrane and the Golgi apparatus^15^, whereas β2AR can generate cAMP responses from the plasma membrane and endosomes^21^. The significance of generating cAMP from distinct membrane compartments is beginning to be understood. There is evidence for distinct transcriptional responses^22,23^ and activating distinct signaling pathways depending on the subcellular source of cAMP production^6,22,24–27^.

Classically, cAMP was considered a highly diffusible molecule, and thus it was reasoned that cAMP generation at the plasma membrane, by GPCR/Gs activation, is sufficient to activate downstream effectors of cAMP in other subcellular membrane compartments. Recent reports, however, demonstrate that cAMP is mostly immobile and constrained due to binding to specific cAMP binding sites. Several studies have reported the role of phosphodiesterases (PDEs) and the regulatory subunit of protein kinase A (PKA), the main cAMP binding protein, in constraining cAMP at specific membrane compartments^28,29^. In the basal state, cAMP was shown to be mostly bound to intracellular cAMP binding sites, such as PKA regulatory subunits, at each subcellular location and PDEs can generate a nanometer-size domain around a source of cAMP^30,31^. Moreover, the PKA regulatory subunit has been shown to form a liquid–liquid phase in the cytoplasm and sequester cAMP, thereby acting as a sponge to buffer cAMP in the cytoplasm^32^. It is only after the elevation of cAMP in cells, that free cAMP can act on PKA and other effectors to initiate downstream cellular responses. Furthermore, the catalytic activity of PKA has also been shown to be constrained to targets within a radius of 15–25 nm^33^. How this spatially and functionally restricted PKA can phosphorylate downstream targets localized within the cells is unclear. Thus, the nanometer scale of the cAMP diffusion range conflicts with the prevalent model whereby plasma membrane-localized receptors generate cAMP, which then propagates linearly to activate cAMP-mediated PKA responses in distant subcellular locations^34,35^.

The present study reveals the pivotal role of local generation of cAMP in controlling local PKA activation at specific subcellular compartments. We demonstrate how cells with more complex architecture, such as cardiomyocytes, can precisely sense subcellular cAMP pools and regulate local PKA activity to generate compartment-specific cellular and physiological outputs.

To determine the relevance of local cAMP generation and the activity map of cAMP around activated β1AR at distinct membrane locations, we measured the activation of downstream effectors of cAMP/PKA in cardiomyocytes. Using an optogenetic approach, we show that local generation of cAMP at the Golgi leads to distinct activation of downstream effector of PKA that increases the rate of relaxation in cardiomyocytes. Conversely, we demonstrated that activation of the plasma membrane pool of β1AR, using pharmacological and genetic approaches, leads to the activation of proximal PKA effectors at the plasma membrane that increase the force of contraction in cardiomyocytes. Finally, we tested two different animal models, zebrafish and mice, using optogenetic and pharmacological approaches and found distinct regulation of cardiac inotropy and lusitropy by different cAMP pools.

## Result

### An optogenetic system to generate cAMP at the Golgi

**Fig. 1:**
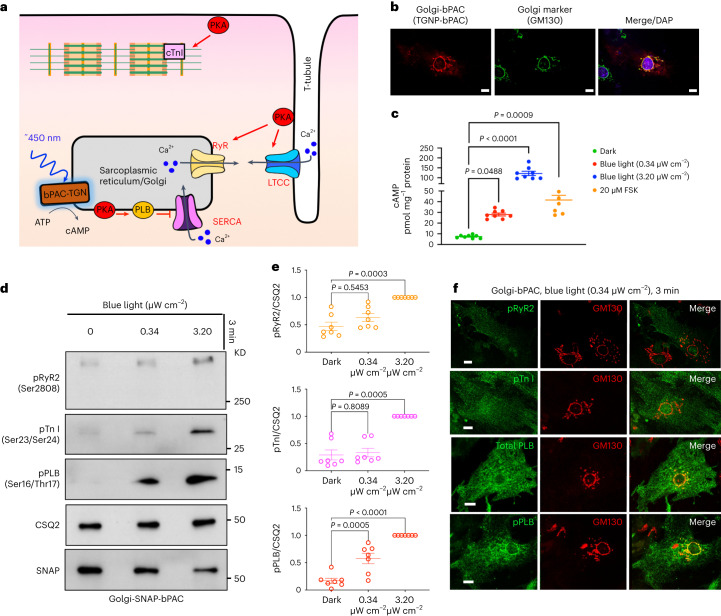
cAMP generation at the Golgi distinctly regulates cardiomyocyte relaxation in neonatal mouse cardiomyocytes. **a**, Illustration of the roles of subcellular cAMP/PKA signaling hubs in regulating cardiomyocytes contraction/relaxation. Blue light stimulation of Golgi-bPAC (TGNP-bPAC) generates cAMP to increase PLB phosphorylation and promote cardiac muscle relaxation **b**, Representative images of Golgi-bPAC (red) and Golgi marker (green), visualized by SNAP and GM130 antibodies and DAPI staining. Scale bar = 10 μm. *n* = 14, 2 biological replicates **c**, cAMP generation mediated by Golgi-bPAC in neonatal cardiomyocytes. Cells were stimulated with blue light for 3 min and 5 min or FSK (20 μM) for 5 min and then lysed for direct cAMP determination by ELISA. cAMP concentrations were normalized to the relative protein concentrations in the cell lysate of each sample. The quantified data are represented as mean ± s.e.m. The *P* values were calculated by one-way ANOVA. *n* = 8 and 6 biological replicates form Golgi-bPAC and FSK-treated cardiomyocytes, respectively. **d**, Representative phosphorylation profiles of RyR2, TnI and PLB induced by Golgi-bPAC in mouse neonatal cardiomyocytes. The protein levels of pRyR2 Ser2808, pTnI Ser23/Ser24 and pPLB Ser16/Thr17 were analyzed in the Golgi-bPAC-expressing mouse neonatal cardiomyocytes kept in the dark or exposed to 0.34 μW cm^−^^2^ or 3.2 μW cm^−^^2^ blue light for 3 min. The protein level of Golgi-bPAC was analyzed using the SNAP antibody. The protein level of CSQ2 was used as a loading control. **e**, The band intensities of pRyR2, pTnI and pPLB were normalized to CSQ2 intensity and then reported as a percentage of the highest value in the groups. The quantified data from different experiments are presented as mean ± s.e.m. The *P* values were calculated by one-way ANOVA. *n* = 7 biological replicates. **f**, The subcellular localization of pRyR2, pTnI, PLB and pPLB upon stimulation of Golgi-bPAC with 0.34 μW cm^−^^2^ blue light in mouse neonatal cardiomyocytes. The protein localizations of pRyR2 Ser2808, pTnI Ser23/Ser24, total PLB and pPLB Ser16/Thr17 antibodies were visualized (green) with Golgi marker stained by GM130 (red). *n* = 25, 3 biological replicates. Scale bar = 10 μm. Source data

**Extended Data Fig. 1:**
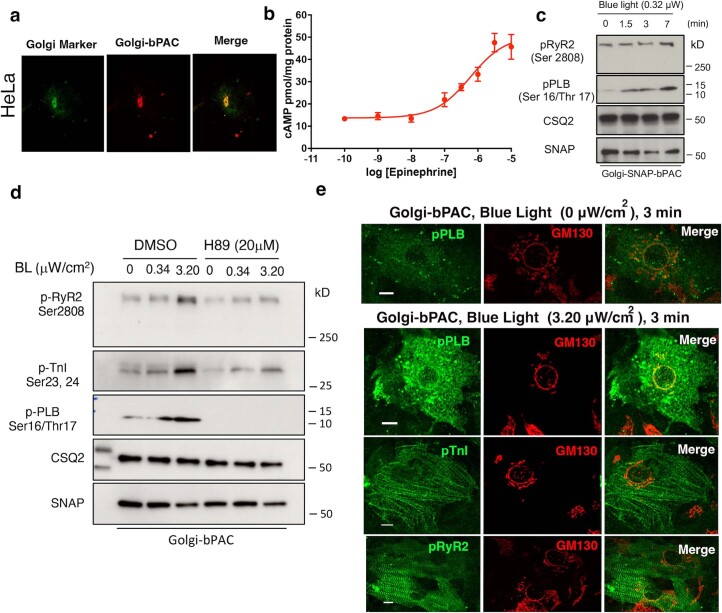
Golgi-bPAC stimulates cAMP generation in response to blue stimulation over time. **a**. Representative images of HeLa cells expressing Golgi-bPAC. n = 36, 6 biological replicates. **b**. Concentration-response curve of cAMP generation in mouse neonatal cardiomyocytes upon various epinephrine concentrations n = 4 biological replicates. The quantified data are represented as mean ± S.E.M. **c**. Phosphorylation profiles of RyR2 and PLB induced by Golgi-bPAC in mouse neonatal cardiomyocytes. The protein levels of p-RyR2 Ser2808 and p-PLB Ser16/Thr17 were analyzed in the Golgi-bPAC-expressing mouse neonatal cardiomyocytes kept in the dark or exposed to 0.34 or 3.20 μW/cm^2^ blue light for 1.5, 3 and 7 minutes. n = 1. **d**. Phosphorylation profiles of RyR2, TnI and PLB induced by Golgi-bPAC before and after treatment of 20μM PKA inhibitor (H89) in mouse neonatal cardiomyocytes. The phospho protein levels were detected using of p-RyR2 Ser2808, p-TnI Ser23/24 and p-PLB Ser16/Thr17 antibodies. The protein level of Golgi-bPAC was analyzed using the SNAP antibody. The protein level of CSQ2 was used as a loading control. n = 1. **e**. The subcellular localization of p-PLB before blue light illumination (top row); the subcellular localization of p-PLB p-RyR2, p-TnI, upon stimulation of Golgi-bPAC with 3.20 μW/cm^2^ blue light in mouse neonatal cardiomyocytes (bottom rows). The protein localizations of p-RyR2 Ser2808, p-TnI Ser23/24, total PLB, and pPLB Ser16/Thr17 antibody were visualized (green) with Golgi marker stained by GM130 (red). n = 27, 3 biological replicates. Scale bar, 10 μm. Source data

To assess whether cAMP generation from the Golgi membrane communicates different cellular information, we developed an optogenetic system based on bacterial photo-activatable adenylyl cyclase (bPAC). bPAC had been previously used to generate cAMP from distinct cellular compartments such as endosomes and cilia^23,36^. We fused bPAC to the *trans*-Golgi network 46 protein, a known Golgi-targeting motif, to target bPAC to the *trans*-Golgi membrane (Extended Data Fig. 1a). We also confirmed Golgi localization in cardiomyocytes (Fig. 1a,b). To assess whether cardiomyocytes expressing Golgi-bPAC generate cAMP in response to blue light treatment, we virally transduced neonatal cardiomyocytes and measured cAMP concentrations. Treating the cells with 0.34 μW cm^−^^2^ blue light for 3 min resulted in ~20 pmol mg^−^^1^ cAMP accumulation in cardiomyocytes (Fig. 1c). This is consistent with the physiological levels of cAMP in cardiomyocytes in response to β1AR stimulation with 100 nM epinephrine (Extended Data Fig. 1b). Considering that the average volume of cardiomyocytes is reported at around 15 picolitres (15,000 μm^3^)^37^, this concentration translates into ~1 μM cAMP in each cell, which is within the physiological levels of cAMP upon hormone stimulation^30,38^.

Because cAMP generated from activated β1AR results in the activation of PKA in cardiomyocytes, we investigated whether cAMP generation from the Golgi membrane can activate downstream targets of PKA. In cardiomyocytes, β1AR-mediated cAMP generation regulates chronotropy (heart rate), inotropy (force of contraction) and lusitropy (relaxation) through PKA-mediated phosphorylation of proteins, such as cardiac troponin I (TnI), ryanodine 2 receptors (RyR2) and phospholamban (PLB; Fig. 1a)^11^. Thus, we examined whether generating physiological levels of cAMP by Golgi-bPAC can phosphorylate and activate downstream targets of PKA. Golgi-bPAC expression in the absence of blue light had minimal effect on the phosphorylation of PKA effectors (Fig. 1d, left lanes). Notably, 0.34 μW cm^−^^2^ blue light treatment resulted in the robust phosphorylation of PLB but not TnI and RyR2 (Fig. 1d, middle lane and quantification in Fig. 1e). This suggests that cardiomyocytes selectively respond to physiologically relevant Golgi-generated cAMP levels. Consistent with this, subcellularly localized PDEs have been shown to constrain cAMP levels to the vicinity of its site of generation^28–30,39,40^.

We then explored whether supraphysiological cAMP levels can overcome this selective response to Golgi-generated cAMP. We manipulated two variables, blue light intensity and exposure time, to reach cAMP levels that are similar to physiological and supraphysiological levels. By increasing blue light intensity to 3.20 μW cm^−^^2^ for 3 min, we found that Golgi-generated cAMP reaches supraphysiological levels and causes the phosphorylation of all three PKA effectors (Fig. 1c,d, right lanes and quantification in Fig. 1e). We then tested the consequences of keeping the blue light intensity low (0.34 μW cm^−^^2^) but increasing the exposure time. As in the case of increased intensity, a 7-min exposure time also led to the phosphorylation of all effectors (Extended Data Fig. 1c). We further confirmed that phosphorylation of PLB, TnI and RyR2 by Golgi-bPAC is mediated by PKA, as inhibiting PKA activity using PKA inhibitor (H89) diminished phosphorylation of all three effectors (Extended Data Fig. 1d). These results suggest that differential consequences of cAMP generated at the Golgi are mediated by PKA and that at physiological levels, only PLB, a regulator of lusitropy, is phosphorylated.

PLB is a sarco/endoplasmic reticulum (SR)-localized protein and is the dominant regulator of Ca^2+^ reuptake by sarco/endoplasmic reticulum Ca^2+^ ATPases (SERCA)^41^. We, therefore, asked whether the local pool of cAMP that is generated by Golgi-bPAC phosphorylates PLB in the vicinity of the Golgi membrane. Immunofluorescence imaging of the nonphosphorylated form of PLB in neonatal cardiomyocytes revealed an SR-localization pattern throughout the cytoplasm, as expected (Fig. 1f, third row). The fluorescent signals were not detectable when we used antibodies against the phosphorylated form of PLB in cardiomyocytes that were unstimulated by blue light (Extended Data Fig. 1e, top row). When cardiomyocytes were stimulated with 0.34 μW cm^−^^2^ blue light for 3 min, immunofluorescence imaging of phosphorylated PLB (pPLB) detected the distribution of pPLB in the vicinity of the Golgi membranes (Fig. 1f, bottom row). The fluorescent signals were not detectable when we used antibodies against the phosphorylated forms of Tn-I and RyR2 at this blue light regimen (Fig. 1f, top rows). However, as we increased cAMP generation by 3.20 μW cm^−^^2^ blue light stimulation for 3 min, phosphorylated forms of all three effectors were detected (Extended Data Fig. 1e). Notably, at this higher cAMP concentration, pPLB no longer had a near Golgi distribution but showed a broad SR-localization pattern (Extended Data Fig. 1e, second row). Together, these data suggest that cAMP generation from the Golgi membranes at similar levels as those generated by sympathetic hormones results in PKA-mediated phosphorylation of the downstream target, PLB, in the vicinity of the Golgi membranes.

### Golgi-cAMP regulates cardiac relaxation in zebrafish

**Fig. 2:**
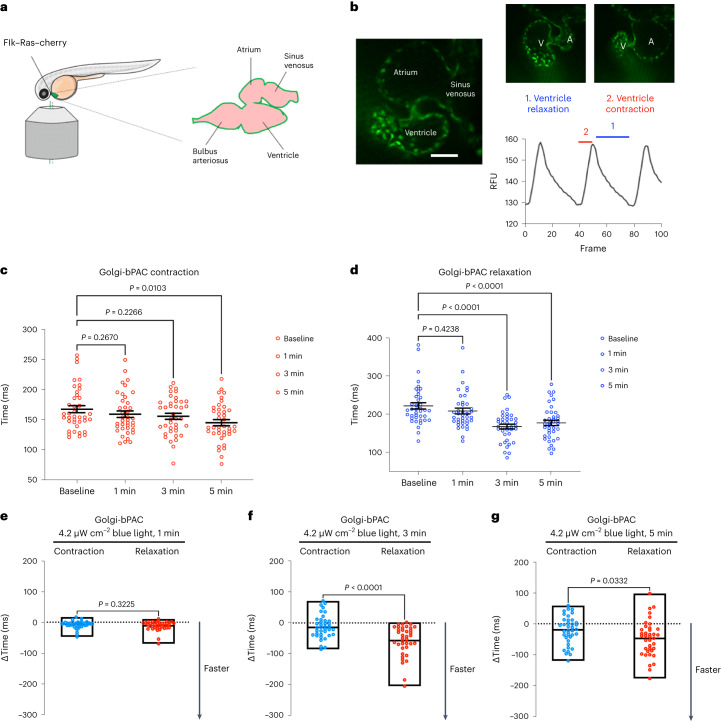
Golgi-delimited cAMP generation promotes faster ventricular relaxation in zebrafish. **a**, Illustration of the mounting position of the zebrafish to image the heart and diagram of the zebrafish heart. **b**, Representative image of a live zebrafish heart (left), demonstrating ventricular contraction and relaxation (right). Fluctuations in the fluorescence of the heart during contraction and relaxation over time are measured. The time between fluorescence maxima to minima is the time of relaxation (1), and the subsequent fluorescence minima to maxima portion of the graph is measured as the time of contraction (2). *n* = 38, 6 biological replicates. Sale bar, 50 μm. A, atrium; V, ventricle. **c**, Changes in heart contraction. **d**, Relaxation time relative to baseline in Golgi-bPAC-expressing zebrafish. Basal images of the zebrafish hearts were acquired for 1,500 frames. The transgenic Golgi-bPAC zebrafish were exposed to 4.2 μW cm^−^^2^ blue light and imaged after 1, 3 and 5 min stimulation. The quantified data are represented as mean ± s.e.m. The *P* values were calculated by one-way ANOVA. *n* = 38 zebrafish, 6 biological replicates. **e**–**g**, Comparisons of rates of contraction and relaxation at 1, 3 and 5 min after 4.2 μW cm^−^^2^ blue light illumination. The differences between faster contraction versus relaxation time over baseline are quantified and presented here as floating bars indicating minimum and maximum with line at median with *P* values presented. Data were analyzed by two-tailed *t*-test. *n* = 38 zebrafish, 6 biological replicates. Source data

**Extended Data Fig. 2:**
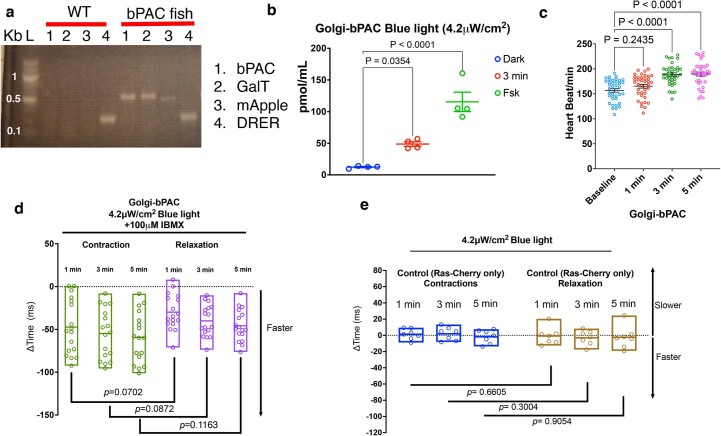
Contraction and relaxation responses of Control Tg(Flk:Ras-cherry)s896 zebrafish to blue light stimulation. **a**. Representative agarose gel electrophoresis of wild-type (WT) and GalT-bPAC-mApple zebrafish genotyping. DNA fragments of GalT, bPAC, and mApple, were amplified from caudal fin samples to sort the transgenic fish. DRER is the DNA extraction and genotyping control for ZF samples. n = 6 biological replicate **b**. Golgi-bPAC zebrafish (72hpf) generate cAMP in response to 3 minutes blue light (4.2 μW/cm^2^). 100 μM FSK treated zebrafish was used as a positive control. The quantified data from different experiments are presented as mean ± S.E.M. The p-values were calculated by one-way ANOVA. n = 4 biological replicates. **c**. Heart rate measurements of Golgi-bPAC zebrafish in response to 1, 3, and 5 minutes blue light (4.2 μW/cm^2^). The quantified data from different experiments are presented as mean ± S.E.M. The p-values were calculated by one-way ANOVA. n = 38 zebrafish **d**. Golgi-bPAC zebrafish were pretreated with 100 μM IBMX for 90 minutes and then exposed to 4.2 μW/cm^2^ blue light at each time points. The differences between faster contraction versus relaxation time over baseline are quantified and presented here as floating bars indicating minimum and maximum with line at median. p-values were calculated by two-tailed t-test. n = 17 zebrafish, 3 biological replicates. **e**. Control (Tg(Flk:Ras-cherry)) zebrafish were stimulated with 4.2 μW/cm^2^ blue light for 1, 3, and 5 min and changes in time of contraction and relaxation were calculated. The differences between faster contraction versus relaxation time over baseline are quantified and presented here as floating bars indicating minimum and maximum with line at median. p-values were calculated by two-tailed t-test. n = 7 control Flk:Rash-cherry zebrafish, 3 biological replicates. Source data

As PLB is the key regulator of cardiomyocyte relaxation (lusitropy), we predicted that cAMP generation at the Golgi specifically regulates lusitropy. To test this hypothesis, we generated a Golgi-bPAC-expressing transgenic zebrafish. Zebrafish is a well-established animal model for exploring the physiological parameters of cardiac function. The molecular mechanisms underlying their heart function are very similar to those of higher vertebrates^42^. The optical clarity of zebrafish embryos allows the real-time and in vivo visualization of the heart contractility responses and makes it a useful vertebrate model system for studying cardiovascular performance using optogenetic tools^43^. To measure cardiac outputs such as chronotropy, inotropy and lusitropy, we generated transgenic zebrafish that express Golgi-bPAC (Extended Data Fig. 2a). These transgenic zebrafish were developed from the established line, Tg(Flk:Ras-cherry)^s896^, which expresses Ras-Cherry in the inner layer of the heart wall (endocardium)^44^. As a result, we were able to trace the motion of the walls of the cardiac atrium (A) and ventricle (V) in different phases of the cardiac cycle in the red channel using a confocal microscope imaging mCherry (Fig. 2a,b). The coupling of ventricular and atrial contraction can be determined by evaluating the time delay between the peak values of the extracted synchronous chronologies within the same cardiac cycle (Fig. 2b). We were also able to measure cardiac rhythm (heart rate) by measuring the distance between two consecutive highest points of the peaks of each cycle.

Treating Golgi-bPAC-expressing zebrafish with 4.2 μW cm^−^^2^ blue light for 3 min resulted in detectable levels of cAMP in zebrafish (Extended Data Fig. 2b). To test the effect of cAMP accumulation in zebrafish hearts, we first measured their heart rate in 72 h postfertilization (hpf). The basal heart rate of zebrafish is around 120–180 beats per minute. Illuminating Golgi-bPAC zebrafish with 4.2 μW cm^−^^2^ blue light resulted in an increase in the heart rate in a time-dependent manner (Extended Data Fig. 2c). Because the heart rate measurement using this assay is determined by adding the rate of contraction to the rate of relaxation, we sought to specifically evaluate each rate at a given time upon blue light illumination. Treating Golgi-bPAC-expressing zebrafish with 4.2 μW cm^−^^2^ blue light for 1 min did not generate measurable cAMP level and had no substantial effect on the heart rate, the mean rate of relaxation (lusitropy) or the contraction (inotropy; Fig. 2c–e). Increasing the blue light illumination time to 3 min also did not result in a substantial change in the mean rate of contraction but increased the mean rate of relaxation (Fig. 2c,d). Illuminating blue light for 5 min resulted in a substantial change in the mean rate of contraction (inotropy; Fig. 2c) and relaxation (lusitropy; Fig. 2d). Notably, the rate of relaxation increased more substantially when compared to the rate of contraction time at 3 min (Fig. 2f). This result is consistent with the specific phosphorylation of PLB but not TnI and RyR2 in cardiomyocytes using low-level blue light treatment. However, at 5 min blue light illumination, the rate of relaxation and contraction increased similarly (Fig. 2g).

Treating Golgi-bPAC zebrafish with 4.2 μW cm^−^^2^ blue light and the PDE inhibitor, 3-isobutyl-1-methylxanthine (IBMX), to increase cAMP levels, elevated the rate of both contraction and relaxation at all time points (Extended Data Fig. 2d). Notably, stimulating control zebrafish (Tg(Flk:Ras-cherry)^s896^) with 4.2 μW cm^−^^2^ blue light at 1, 3 and 5 min did not result in substantial changes in the rate of contraction or relaxation (Extended Data Fig. 2e). Altogether, these results suggest that cAMP generation from the Golgi at physiological levels specifically regulates the rate of cardiac relaxation responses. However, this differential interpretation of cAMP is disrupted when cAMP generation at the Golgi is increased to supraphysiological levels and PDEs are no longer able to constrain cAMP to the vicinity of a compartment^28,40^.

### Plasma membrane and Golgi pools of β1ARs function differently

**Extended Data Fig. 3:**
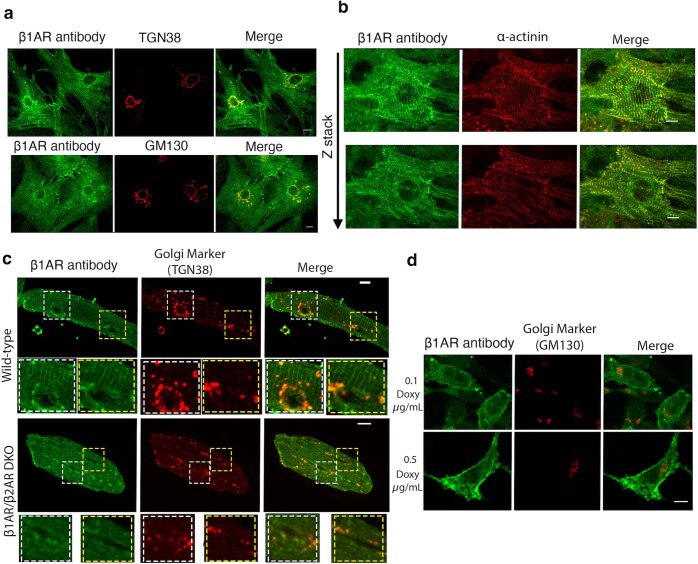
Plasma membrane and Golgi localization of β1AR in mouse neonatal and adult cardiomyocytes. **a**. Representative images of β1AR (green) localization relative to TGN38 (trans-Golgi) and GM130 (cis-Golgi) in mouse neonatal cardiomyocytes. n = 31, 3 biological replicates **b**. Representative images of β1AR (green) localization relative to α-actinin (z-disk marker) in mouse neonatal cardiomyocytes. Endogenous β1AR is expressed on the plasma membrane and the Golgi membranes. n = 25, 3 biological replicates **c**. Isolated adult cardiomyocytes from wild-type showed distinct t-tubule and Golgi localization, as detected by β1AR (green) and TGN38 (red) (Golgi marker) antibodies. The distinct t-tubules and Golgi staining of β1AR are not detectable in adult cardiomyocytes isolated from β1AR/β2AR double knock-out mice. n = 16, 3 biological replicates **d**. Representative images of HeLa cells transfected with a doxycycline-inducible promotor (Tet-on) controlled β1AR construct, treated with 0.1 and 0.5 μg/ml doxycycline for 24 hours post transfection. β1AR (green) was detected using β1AR antibody. n = 37, 3 biological replicates. Scale bar, 10 μm. Source data

Given that Golgi-generated cAMP specifically regulates PLB phosphorylation and lusitropy, we hypothesized that hormone-mediated cAMP responses by Golgi-localized β1AR should regulate cAMP-mediated PLB phosphorylation. Moreover, we hypothesized that β1ARs at the plasma membrane control downstream effectors in the vicinity of the plasma membrane. β1ARs localize to both the plasma membrane and Golgi membranes of neonatal and adult cardiomyocytes, as detected by immunostaining using an antibody against β1AR (Extended Data Fig. 3a–c). Interestingly, although we observed β1AR staining on both the plasmalemma and t-tubules in adult cardiomyocytes, as reported previously^45,46^, β1AR staining was mainly detected on the striated pattern on the plasma membrane and not detectable on the plasmalemma in neonatal cardiomyocytes (Extended Data Fig. 3a,b). This may reflect the developmental stage differences between neonatal and adult cardiomyocytes. We have previously confirmed the specificity of the antibody using two different siRNAs against β1AR^15^. We further confirmed this immunostaining result using adult cardiomyocytes derived from β1AR/β2AR double-knockout mice. We did not detect any fluorescence signal in either the Golgi or the plasma membrane in β1AR/β2AR double-knockout cardiomyocytes (Extended Data Fig. 3c). We then tested whether this antibody could detect low levels of β1AR in transfected cell lines. HeLa cells were transfected with doxycycline-inducible promotor (Tet-on) β1AR construct and were treated with 0.1 μg ml^−1^ and 0.5 μg ml^−1^ doxycycline for 24 h post transfection. Low levels of β1AR expressing cells were detected using the β1AR antibody used here (Extended Data Fig. 3d), further confirming the specificity of this antibody.

**Extended Data Fig. 4:**
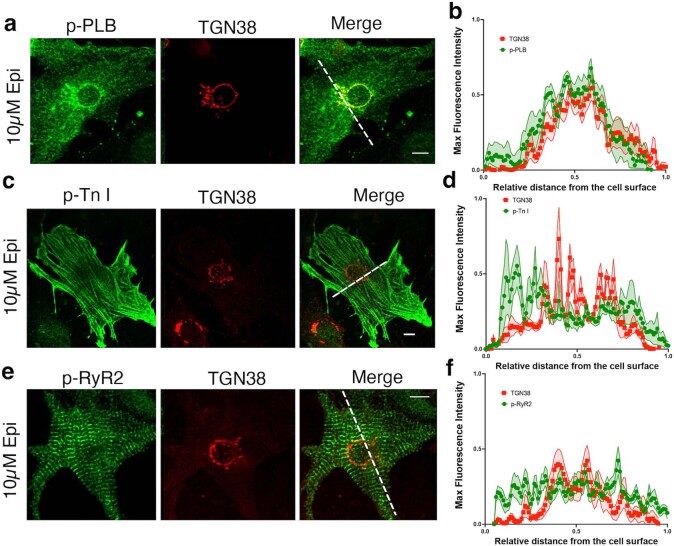
Epinephrine stimulation of mouse neonatal cardiomyocytes reveals spatially distinct phosphorylation patterns of downstream PKA effectors. **a-f**. Representative images of epinephrine-mediated phosphorylation of PLB, TnI, and RyR2 in neonatal cardiomyocytes. Neonatal cardiomyocytes were incubated with epinephrine (10 μM) for 20 minutes and immune-stained for p-RyR2 Ser2808, p-TnI Ser23/24, or p-PLB Ser16/Thr17, and TGN38. Representative ROIs in the merged images were analyzed by fluorescence line scan intensity and shown in the corresponding graphs. The maximal fluorescence intensity of the pPLB, pTnI, and pRyR2, relative to the Golgi markers, are measured along the width of the neonatal cardiomyocytes. The length of each ROI was normalized and organized into 100 bins; the average intensity of each bin is shown. These graphs demonstrate the phosphorylated proteins’ localization, spread, and intensity throughout the cells. n = 12, 4, 12 cells for pPLB, pTnI and pRyR2, respectively. 3 biological replicates. Scale bar, 10 μm. Source data

We previously reported that both the plasma membrane and the Golgi pool of β1ARs can promote cAMP generation^6,15^. In healthy cardiomyocytes, β1AR signaling regulates cardiac responses through PKA-mediated phosphorylation of proteins such as TnI, RyR2 and PLB^11^. We used immunofluorescence imaging to visualize cellular localization patterns of phosphorylated forms of RyR2, TnI and PLB upon 10 μM epinephrine in cardiomyocytes. Although pPLB localized near the perinuclear/Golgi membranes (Extended Data Fig. 4a,b), phosphorylated TnI and RyR2 did not colocalize with the Golgi marker and showed a plasmalemma (the outer plasma membrane regions in cardiomyocytes) and striated localization pattern on the plasma membrane, respectively (Extended Data Fig. 4c–f). Based on this distinct localization pattern, we hypothesized that β1AR-mediated cAMP likely regulates distinct PKA effectors in each membrane compartment’s vicinity.

**Fig. 3:**
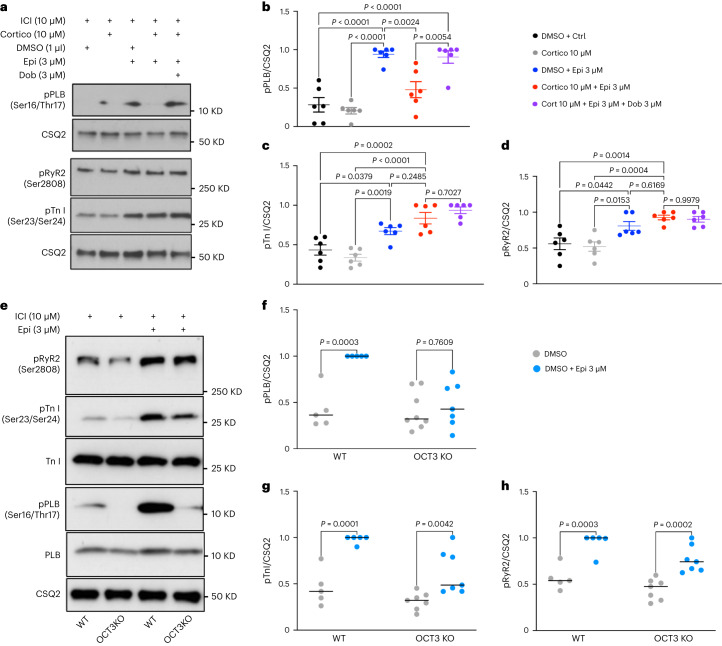
Plasma membrane and the Golgi pools of β1AR function differently in adult mouse cardiomyocytes. **a**, Representative western blots of phosphorylation profiles of RyR2, TnI and PLB regulated by β1AR in adult cardiomyocytes derived from WT mice, treated with 10 μM corticosterone (Cortico). Adult cardiomyocytes were pretreated with 10 μM β2AR-selective antagonist ICI-118551 (ICI) to isolate the function of β1ARs. Membrane-permeable β1AR-selective agonist, dobutamine (Dob), promotes PLB phosphorylation independent of OCT3. **b**–**d**, Quantification of immunoblots of pRyR2 Ser2808, p-TnI Ser23/Ser24 and pPLB Ser16/Thr17 normalized to the protein level of CSQ2 and then reported as a percentage of the highest value in the groups. The quantified data from different experiments were presented as mean ± s.e.m. The *P* values were calculated by one-way ANOVA. *n* = 6 biological replicates. **e**, Representative western blots of phosphorylation profiles of RyR2, TnI and PLB regulated by β1AR in adult cardiomyocytes derived from OCT3 (*SLC22A3*) knockout mice and compared to WT. **f**–**h**, Quantification of immunoblots of pRyR2 Ser2808, pTnI Ser23/Ser24 and pPLB Ser16/Thr17 normalized to the protein level of CSQ2 and then reported as a percentage of the highest value in the groups. The quantified data from different experiments were presented as mean ± s.e.m. The *P* values were calculated by two-way ANOVA. *n* = 5 and 7 biological replicates for WT and OCT3 knockout cardiomyocytes, respectively. Source data

**Extended Data Fig. 5:**
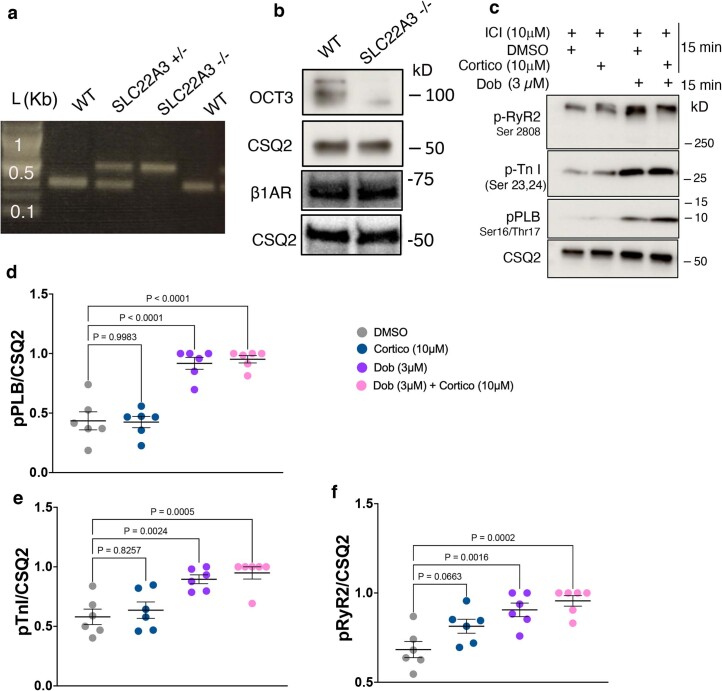
Membrane permeable β1AR selective agonist, dobutamine, promotes PLB phosphorylation independent of OCT3 inhibition by corticosterone. **a**. Representative agarose gel electrophoresis for genotyping of wild-type, heterozygous, and homologous OCT3 (*SLC22A3*) knock-out mice. n = 7 biological replicates **b**. Western blot of OCT3 (*SLC22A3*) (top) and β1AR (bottom) and CSQ2 from wild-type and OCT3 (*SLC22A3*) knock-out-derived cardiomyocytes. n = 3 biological replicates **c**. Representative western blots of adult cardiomyocytes pretreated with 10 μM corticosterone (cortico) and 10 μM ICI-118551 (ICI), then stimulated with 3 μM dobutamine (Dob). **d-f**. Quantification of Western blot of p-RyR2 Ser2808, p-TnI Ser23/24, and p-PLB Ser16/Thr17 normalized to the protein level of CSQ2, and then reported as a percentage of the highest value in the groups. The quantified data from different experiments were presented as mean ± S.E.M. The p-values were calculated by two-way ANOVA. n = 6 biological replicates. Source data

**Extended Data Fig. 6:**
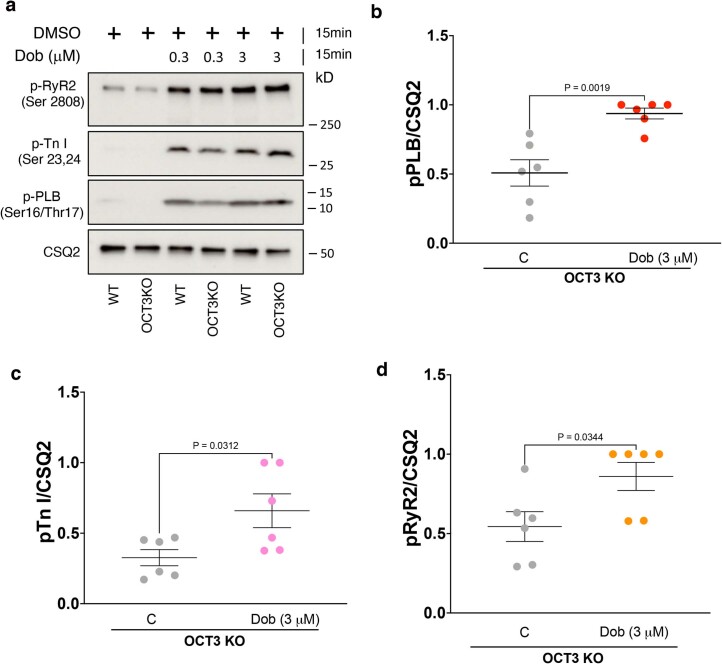
Membrane permeable β1AR selective agonist, dobutamine, promotes PLB phosphorylation in adult OCT3 knock-out cardiomyocytes. **a**. Representative western blots of adult cardiomyocytes derived from wild-type or OCT3 knock-out stimulated with 0.3 or 3 μM dobutamine (Dob). **b-d**. Quantification of Western blot of p-RyR2 Ser2808, p-TnI Ser23/24, and p-PLB Ser16/Thr17 normalized to the protein level of CSQ2, and then reported as a percentage of the highest value in the groups. The quantified data from different experiments were presented as mean ± S.E.M. The p-values were calculated by two-tailed t test. n = 6 biological replicates. Source data

To assess whether the plasma membrane and Golgi pools of β1AR regulate different PKA effectors, we pharmacologically blocked β2AR with 10 μM ICI-118551 to specifically test the function of β1AR in cardiomyocytes. We took advantage of membrane-permeant and impermeant agonists of β1AR to compare the functions of plasma membrane and Golgi-localized β1AR in adult cardiomyocytes. We have previously demonstrated that epinephrine, a membrane-impermeant βAR agonist, requires a monoamine transporter, organic cation transporter 3 (OCT3), to reach the Golgi lumen and activate Golgi-localized β1AR^15^. OCT3 is expressed in cardiomyocytes (Extended Data Fig. 5a,b)^47^. Pharmacological inhibition of OCT3 inhibits epinephrine/norepinephrine-mediated Golgi-localized β1AR activation^6,15^. Notably, OCT3 inhibition abolishes epinephrine-mediated phosphorylation of PLB but not β1AR-mediated phosphorylations of TnI and RyR2 (Fig. 3a–d). Unlike epinephrine, dobutamine, a membrane-permeable β1AR agonist, can activate Golgi-localized β1AR independently of OCT3 (Fig. 3a, last lane, and Extended Data Fig. 5c–f). Additionally, cardiomyocytes derived from OCT3 knockout mice that have similar expression levels of β1AR as wild-type (WT) cardiomyocytes (Extended Data Fig. 5b) showed no PLB phosphorylation upon epinephrine stimulation but displayed an increase in TnI and RyR2 phosphorylation (Fig. 3e–h). In contrast, dobutamine caused phosphorylation of TnI, RyR2 and PLB in OCT3 knockout cardiomyocytes (Extended Data Fig. 6a–d). Thus, our results indicate that plasma membrane-localized β1AR regulates phosphorylations of TnI and RyR2 to control inotropy (contraction), whereas Golgi-localized β1AR regulates PLB phosphorylation to control lusitropy (relaxation).

**Extended Data Fig. 7:**
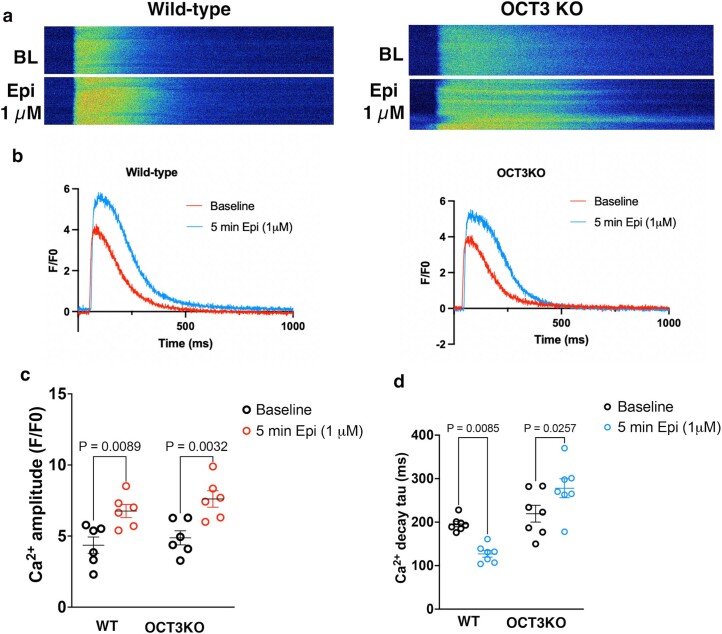
Epinephrine-mediated Ca^2+^ decay tau was delayed in adult cardiomyocytes derived from OCT3 knock-out mice. **a**. Adult cardiomyocytes were loaded with 5 μM Ca^2+^ indicator (Fluo-4 AM). Representative line scan images and the corresponding trace of fluorescence obtain from epinephrine-mediated Ca^2+^ response upon 1 Hz field stimulation in adult cardiomyocytes derived from wild-type (left) and OCT3 knock-out mice (right). **b, c**. Ca^2+^ transient amplitude, and **d**. decay tau were recorded with 1 Hz pacing and upon 1 μM epinephrine stimulation for 5 minutes in adult cardiomyocytes derived from wild-type and OCT3 knock-out mice. The quantified data from different experiments were presented as mean ± S.E.M. The p-values were calculated by two-way ANOVA. n = 6 and 7 cells for amplitude and decay tau, respectively, 3 biological replicates. Source data

Given that PLB is the key regulator of Ca^2+^ reuptake by the SERCA channel, we then assessed Ca^2+^ dynamics in cardiomyocytes. We isolated adult cardiomyocytes and incubated them with Fluo-4 acetoxymethyl (Fluo-4 AM), a Ca^2+^ dye. Upon 1 Hz field stimulation, both cardiomyocytes derived from the WT and OCT3 knockout showed similar baseline Ca^2+^ dynamics (Extended Data Fig 7a,b). Epinephrine treatment increased the Ca^2+^ transient amplitude in both WT and OCT3 knockout cardiomyocytes (Extended Data Fig. 7c). WT adult cardiomyocytes also showed a decreased calcium decay tau in response to epinephrine stimulation, a readout of accelerated Ca^2+^ reuptake by SERCA upon PLB phosphorylation. However, adult cardiomyocytes isolated from OCT3 knockout mice did not show a substantial decrease in calcium decay tau upon epinephrine treatment (Extended Data Fig. 7d). These results are consistent with the lack of PLB phosphorylation observed in OCT3 knockout cardiomyocytes upon epinephrine stimulation (Fig. 3e,f).

### β1AR autoantibodies activate plasma membrane pools of β1AR

**Fig. 4:**
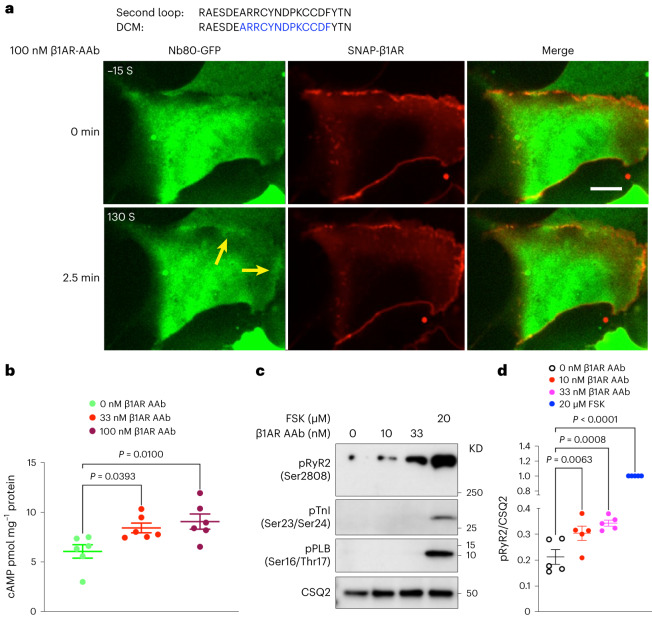
β1AR autoantibody specifically activates plasma membrane-localized β1AR and regulates RyR2 phosphorylation in neonatal mouse cardiomyocytes. **a**, The amino acid similarity between the second extracellular loop of β1AR and an autoantibody (AAb) epitope region (blue color labeled) found in patients with dilated cardiomyopathy (DCM; top), representative images of HEK293 cells transfected with SNAP-tagged β1AR and nanobody-based biosensor for βARs (Nb80-GFP) before and after 100 nM AAb stimulation (bottom). Nb80-GFP is recruited to the plasma membrane-localized β1AR after 2.5 min (arrowheads). *n* = 35, 3 biological replicates, Scale bar = 10 μm. **b**, cAMP generation mediated upon 33 nM and 100 nM AAb treatment in neonatal cardiomyocytes. Cells were stimulated for 15 min and lysed for direct cAMP determination by ELISA. cAMP concentrations were normalized to the relative protein concentrations in the cell lysate of each sample. The quantified data are represented as mean ± s.e.m. The *P* values were calculated by one-way ANOVA, C, control group. *n* = 6 biological replicates. **c**, Representative western blots of RyR2, TnI and PLB phosphorylation profiles regulated by β1AR AAb in mouse neonatal cardiomyocytes. Mouse neonatal cardiomyocytes were treated with 10 nM and 33 nM β1AR AAb or 20 μM FSK for 15 min. **d**, The protein levels of pRyR2 Ser2808, pTnI Ser23/Ser24 and pPLB Ser16/Thr17 were analyzed. The protein level of pRyR2 was normalized with the protein level of CSQ2 and then reported as a percentage of the highest value in the FSK-treated group (20 μM). The quantified data from different experiments were presented as mean ± s.e.m. The *P* values were calculated by one-way ANOVA. *n* = 5 biological replicates. Source data

**Extended Data Fig. 8:**
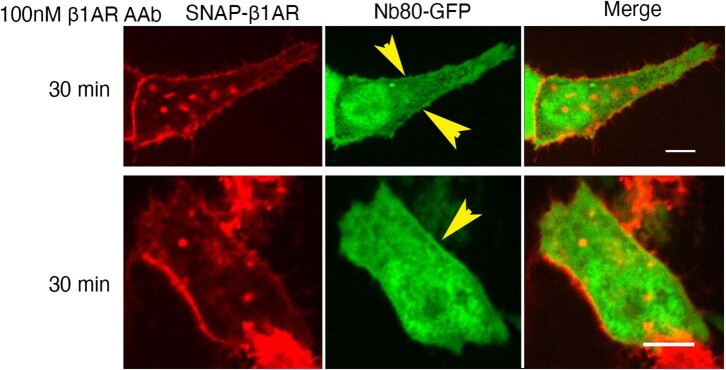
Nb80-GFP is recruited to activated β1AR on the plasma membrane upon autoantibody stimulation. Representative images of HEK293 cells transfected with SNAP-tagged β1AR and nanobody based biosensor for βARs (Nb80-GFP) upon 100 nM AAb stimulation (bottom). Nb80-GFP is recruited to the plasma membrane-localized β1AR after 30 minutes (arrowheads). n = 35, 3 biological replicates. Scare bar, 10 μm. Source data

To further distinguish the roles of Golgi and plasma membrane-β1AR signaling in regulating cardiomyocyte contractility, we took advantage of an autoantibody against β1AR to specifically activate β1ARs only at the plasma membrane. Autoantibodies against β1ARs have been reported in various cardiac diseases, including dilated cardiomyopathy^48,49^. Many of these autoantibodies function as agonists because their epitope sequences have sequence similarities to the extracellular loop 2 of β1ARs (Fig. 4a)^50^. Previous studies have shown a measurable cAMP production and positive inotropic response upon treating cardiomyocytes with autoantibodies^51,52^. Given that antibodies are membrane impermeant and cannot cross the plasma membrane, we used them to activate the plasma membrane pool of β1AR specifically. We first tested whether this antibody functions as an agonist. To do this, we used a previously generated nanobody-based biosensor (Nb80-green fluorescent protein (GFP)) to detect active conformation of βARs^15,21^. Stimulating HEK293 cells expressing SNAP-tagged β1AR and Nb80-GFP with 100 nM antibody generated against extracellular loop 2 of β1AR resulted in subtle recruitment of Nb80-GFP to the plasma membrane-localized β1AR (Fig. 4a and Extended Data Fig. 8), suggesting that the β1AR antibody functions as a partial agonist. Additionally, we found a slight increase in cAMP concentration (~10 pmol mg^−^^1^) when neonatal cardiomyocytes were treated with β1AR autoantibody, further supporting the notion that this antibody acts as a partial agonist (Fig. 4b). We then stimulated isolated cardiomyocytes with two different concentrations of the autoantibody (10 and 33 nM) and found an increased RyR2 phosphorylation but not PLB and TnI phosphorylation (Fig. 4c,d). These data further support a model where different pools of β1ARs regulate distinct functions in cardiomyocytes, with Golgi-localized-β1ARs regulating PLB phosphorylation and the plasma membrane-localized β1ARs regulating RyR2 phosphorylation. Interestingly, we did not observe TnI phosphorylation by β1ARs autoantibody at these concentrations. We speculate that the lack of TnI phosphorylation could be due to the fact that the β1AR autoantibody acts as a partial agonist and generates low levels of cAMP by the activated β1AR on the specific domain on the plasma membrane seen as a striated pattern. This region appears to be in the proximity of RyR2 but not other PKA effectors, such as TnI and PLB that are more distant from that membrane domain (Extended Data Fig. 3b–d,f).

### Plasma membrane and Golgi PKAs have distinct functions

**Fig. 5:**
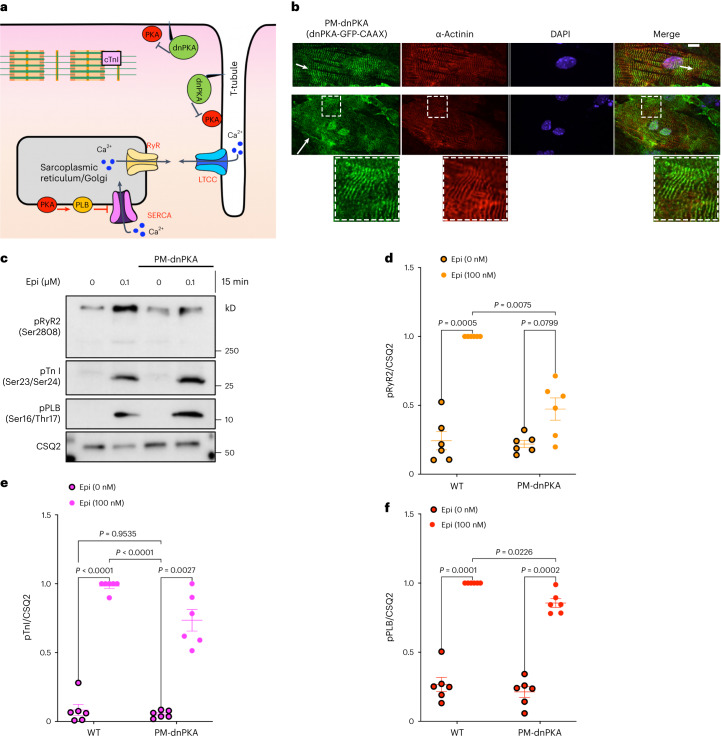
Plasma membrane and the Golgi pool of PKA have distinct functions. **a**, Model of targeting a dnPKA to the plasma membrane (PM-dnPKA) to locally regulate PKA activity in neonatal cardiomyocytes. PM-dnPKA was generated by fusing dnPKA with GFP and CAAX motif. **b**, PM-dnPKA, visualized by GFP (green), was costained with α-actinin (red), a marker of sarcomeric z-disk, and DAPI. Insets show PM-dnPKA and α-`actinin colocalization. *n* = 24, 3 biological replicates. Scale bar = 10 μm. **c**, The representative western blots of RyR2, TnI and PLB phosphorylation profiles regulated by epinephrine in the absence or presence of PM-dnPKA expression. The protein levels of pRyR2 Ser2808, pTnI Ser23/Ser24 and pPLB Ser16/Thr17 were analyzed in WT and PM-dnPKA-expressing moue neonatal cardiomyocytes without or with 0.1 μM epinephrine treatment for 15 min. **d**–**f**, The band intensities of pRyR2, pTnI and pPLB were normalized with CSQ2 intensity and then reported as a percentage of the highest value in the groups. The quantified data from different experiments were presented as mean ± s.e.m. The *P* values were calculated by two-way ANOVA. *n* = 6 biological replicates. Source data

PKA is a holoenzyme composed of two regulatory (PKA-R) and two catalytic (PKA-C) subunits anchored to membranes by A-kinase anchoring proteins (AKAPs). As a result, PKA holoenzymes are highly compartmentalized^34,40,53^. It was commonly believed that the PKA-C subunit dissociates from the PKA-R subunit in the presence of excess cAMP, and thus PKA-C can activate downstream effectors localized within the cells. However, recent studies have revealed that the activity of the PKA-C subunit is constrained to targets within a radius of 15–25 nm^33^. Given that this spatially and functionally restricted PKA can only phosphorylate proximal downstream targets in cardiomyocytes, it stands to reason that the plasma membrane-localized receptors are unlikely to be the sole source of cAMP-mediated PKA activation. To test which pool of PKA within the cells regulates the phosphorylation of downstream effectors, we targeted a dominant-negative PKA (dnPKA), a constitutively repressive version of PKA-RIa that is insensitive to cAMP^54,55^ to the plasma membrane (PM-dnPKA; Fig. 5a). To target dnPKA to the plasma membrane, we fused it to the CAAX motif of the K-Ras protein. PM-dnPKA was mainly localized on thin parallel striation that colocalized with sarcomeric z-disk markers (α-actinin), a membrane region in cardiomyocytes that is involved in organizing t-tubules (Fig. 5b, short arrows)^56,57^. We could also observe a fraction of PM-dnPKA on the plasmalemma of the plasma membrane in neonatal cardiomyocytes (Fig. 5b, long arrow). We then assessed how the inhibition of the plasma membrane pool of PKA affects epinephrine-mediated phosphorylation of downstream PKA targets in cardiomyocytes. Stimulation of neonatal cardiomyocytes with epinephrine resulted in the phosphorylation of RyR2, TnI and PLB. Interestingly, epinephrine-stimulated cardiomyocytes expressing PM-dnPKA showed abrogated RyR2 phosphorylation, but TnI and PLB phosphorylations remained unchanged (Fig. 5c–f). This is consistent with the observation that PM-dnPKA is largely concentrated on specific striated domains on the plasma membrane near RyR2. By contrast, only low levels of PM-dnPKA are seen in plasmalemma, which is near to where TnI is localized (Fig. 5b). These data along with our findings using autoantibodies (Fig. 4c) further confirm that β1AR, concentrated on specific striated membrane domain on the plasma membrane, regulates RyR2 near that location

### OCT3 knockout mice have preserved inotropy but delayed lusitropy

**Fig. 6:**
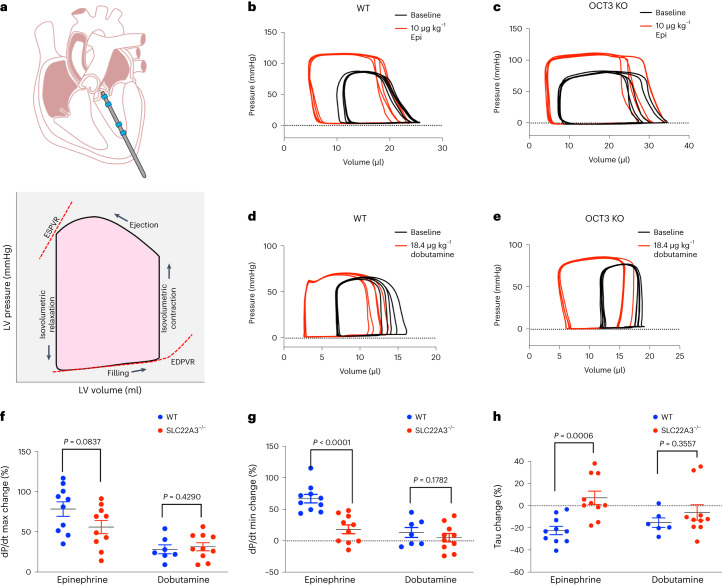
Pressure–volume measurement of OCT3 knockout mice revealed preserved systole but impaired diastole upon epinephrine stimulation. **a**, Diagram demonstrating the placement of the catheter for pressure–volume measurements in mouse hearts (top). Representative pressure–volume loop shows the changes in pressure and volume during isovolumetric contraction. Multiple cardiac indicators, including the end-systolic pressure–volume relationship (ESPVR), end-diastolic pressure–volume relations (EDPVR), stroke volume, cardiac output and ejection fraction, can be derived from PV loops (bottom). **b**–**e**, Representative hemodynamic pressure–volume loops (five loops) upon 10 μg kg^−1^ epinephrine injection in (**b**) WT and (**c**) OCT3 (*SLC22A3*) knockout mice or 18.4 μg kg^−1^ dobutamine injection in (**d**) WT and (**e**) OCT3 knockout mice. **f**, The maximum dP/dt derived from the PV loops (a measurement of systolic function). **g**, The minimum dP/dt (a measurement of diastolic function), upon epinephrine (10 μg kg^−1^) or dobutamine (18.4 μg kg^−1^) bolus injection through the jugular vein of WT and OCT3 (*SLC22A3*) knockout mice. **h**, Tau represents the decay of pressure during isovolumetric relaxation that is preload independent. Data are presented as mean ± s.d. The *P* values were calculated by two-tailed *t*-test. *n* = 10 WT and OCT3 knockout mice for epinephrine treatment and *n* = 7 WT and 10 OCT3 knockout mice for dobutamine treatment. Source data

Increased sympathetic activity during the fight and flight response or exercise causes an increase in epinephrine/norepinephrine levels in the circulation and enhances βARs activity. Thus, the heart efficiently augments cardiac output by increasing the heart rate, dromotropy (conduction speed), inotropy (force of contraction) and lusitropy (rate of relaxation). Our data in isolated adult and neonatal cardiomyocytes suggest that plasma membrane-localized β1AR regulates RyR2 phosphorylation, a key Ca^2+^ channel that increases the release of Ca^2+^ from SR, and thus triggers the cardiac muscle to contract. In contrast, we found that Golgi-localized β1AR specifically regulates PLB phosphorylation, a key regulator of Ca^2+^ reuptake to the SR through SERCA Ca^2+^ channels, thus promoting cardiac muscle relaxation (Figs. 3–5). These data suggest that plasma membrane β1AR regulates inotropy, whereas Golgi-localized β1AR regulates lusitropy. To test this hypothesis, we performed real-time measurements of pressure and volume (PV) loop within the left ventricle of the mice in response to bolus injections of epinephrine (Fig. 6a). Several physiologically relevant hemodynamic parameters, such as stroke volume, ejection fraction, myocardial contractility and lusitropy, can be determined from these loops (Fig. 6a). To measure the PV loop upon stimulation of βARs, we inserted a 1.4-F pressure-conductance catheter and injected mice at the right jugular vein with 10 μg kg^−1^ epinephrine. To isolate the function of the plasma membrane and Golgi-localized β1AR, we compared WT and OCT3 knockout mice (Extended Data Fig. 5a,b)^58^. Given that OCT3 facilitates the transport of epinephrine to the Golgi-localized β1AR and OCT3 inhibition leads to abrogated PLB phosphorylation in cardiomyocytes (Fig. 3e–h), we predicted that OCT3 knockout mice will have delayed lusitropic response. Epinephrine injection induced an increase in the heart rate of both WT and OCT3 knockout mice (Supplementary Table 1). Notably, the maximal rate of left ventricle pressure change (dP/dt max), ejection fraction and cardiac output, which are the key indications of systolic function of contraction (inotropy), were similar upon epinephrine injection between the WT and OCT3 knockout mice (Fig. 6b,c,f and Supplementary Table 1). An increase in contractility is observed as an increase in dP/dt max during isovolumic contraction. Thus, these data suggest that WT and OCT3 knockout mice have a similar rate of contraction upon epinephrine injection. However, the minimal rate of left ventricle pressure change (dP/dt min), which is manifested as an increase in diastolic function or an increase in the rate of relaxation (lusitropy), was delayed in OCT3 knockout mice (Fig. 6c,g and Supplementary Table 1). Moreover, tau, which represents the exponential decay of the ventricular pressure during isovolumic relaxation, was also delayed in OCT3 knockout mice compared to WT (Fig. 6c,h and Supplementary Table 1). Notably, injection of dobutamine, a membrane-permeant β1AR agonist that does not require OCT3 to activate Golgi-localized β1AR^6,15^, caused similar increases in the rate of contraction, relaxation and tau in both WT and OCT3 knockout mice (Fig. 6d–h and Supplementary Table 1). Altogether, these data suggest that OCT3 knockout mice have preserved systole (inotropy) but delayed diastole (lusitropy) upon epinephrine stimulation.

## Discussion

Our findings demonstrate the cellular and physiological significance of cAMP generation at specific locales. We present evidence that cells with more complex architecture, such as cardiomyocytes, distinguish local cAMP generation and elicit different physiological outputs. We demonstrated that localized activation of β1ARs at subcellular compartments leads to local generation of cAMP and activation of downstream PKA effectors that are in the vicinity of each compartment. We found that cAMP generation at the Golgi results in PLB phosphorylation and consequently increased rate of relaxation in cardiomyocytes, an observation verified in the intact hearts of zebrafish. Furthermore, we found that the plasma membrane pool of cAMP regulates local PKA effectors, such as RyR2, leading to increased contractile force. Notably, we showed that a monoamine transporter (OCT3) that facilitates the transport of epinephrine/norepinephrine regulates the activation of Golgi- β1AR-mediated PLB phosphorylation. Thus, epinephrine stimulation in OCT3 knockout cardiomyocytes only activates the β1AR pools at the plasma membrane. This observation was further verified in OCT3 knockout mice, where the force of contraction (systole) was preserved upon epinephrine injection, but the relaxation rate (diastole) was impaired.

Examining the publicly available phenotype across >420,000 individuals in UK Biobank with exome sequencing data shows that loss of function of OCT3 (SLC22A3) is substantially associated with cardiovascular diseases, specifically diastolic pressure^59^. Recently, it was shown that several prescription drugs that potently inhibit OCT3 cause adverse reactions related to cardiovascular traits^60^. The findings from this study suggest the possibility that these adverse reactions may be due to alterations in subcellular cAMP/PKA signaling caused by the inhibition of OCT3.

Our data using an antibody against the extracellular loop 2 of β1AR to specifically activate the plasma membrane pool of β1AR support the notion that β1AR concentrated on the striated domains on the plasma membrane regulate RyR2 phosphorylation but had no effect on phosphorylation of PLB and TnI, which are more distant from that membrane domain. Accordingly, inhibiting PKA in that membrane region in neonatal cardiomyocytes abrogates epinephrine-mediated RyR2 phosphorylation but without substantial effect on TnI and PLB phosphorylation. These findings suggest that the functional pool of β1AR/cAMP/PKA resides on specific striated membrane domains on the plasma membrane, thus regulating the increase in the contractile force of cardiomyocytes. The β1AR autoantibodies are present in more than 30% of patients with dilated cardiomyopathy. It has been reported that these autoantibodies function as an agonist and specifically induce a positive inotropic effect in isolated cardiomyocytes^52^. Our findings provide a potential mechanism for this observation.

There are several different genetically encoded membrane-localized fluorescence and bioluminescence-based biosensors that have been developed to study cAMP compartmentalization^61–64^. Almost all these studies have focused on the role of PDEs, AKAPs and PKA in forming cAMP domains at different subcellular compartments but assumed that the sole source of cAMP are GPCRs that are activated on the plasma membrane. Previous views of localized GPCR signaling have been mainly attributed to receptor-associated cAMP microdomain or nanodomain localization on the plasma membrane. For instance, it has been shown that β2ARs, but not β1ARs, are exclusively associated with caveolae and lipid rafts^65^. Thus, it was thought that the distinct signaling functions of β1AR and β2AR are due to their unique localization on the plasma membrane^45^. More recently, the cAMP nanodomain formation on the plasma membrane has been reported for glucagon-like 1 peptide receptor and β2AR where signaling specificity is determined based on the formation of receptor-associated cAMP nanodomains on the plasma membrane^31^. Although GPCR-mediated cAMP signaling at junctional regions of t-tubules and sarcoplasmic reticulum is well established^62^, the significance of GPCR signaling from intracellular compartments has been mainly explored in the context of signaling from endosomes^14,22,26,66,67^. Nash et al. demonstrated that inhibition of OCT3 abrogates β1AR-mediated Epac-dependent phospholipase Cε activation and hydrolysis of phosphatidylinositol-4-phosphate, a signaling pathway that contributes to the hypertrophic responses^6^. More recently, it has been reported that a pool of β1AR is associated with the SERCA2 complex and regulates calcium transients and contraction responses^24^. Our data here provide evidence for the physiological significance of cAMP nanodomain formation by activated GPCRs on the plasma membrane and the Golgi membranes for regulating distinct cardiac function.

In patients with heart failure, lusitropic effects of catecholamines appear to be exerted by lower concentrations than inotropic effects^68^. It is well established that cAMP compartmentation is disrupted in failing hearts, due to mislocalization of PKA and their corresponding AKAPs that tether PKA to discrete subcellular sites^69,70^. Whether this is due to reduced or enhanced activity of βARs subtypes at specific membrane locations is not known and requires further investigation^68^.

Our new findings on the significance of local cAMP signaling generation by activated β1AR could have important implications for a better understanding of cardiac diseases. For instance, our PV loop measurements of OCT3 knockout mice upon epinephrine stimulation mimic what is seen in diastolic dysfunction, a highly substantial but poorly understood clinical condition where the cardiac muscle contraction is preserved, but relaxation is impaired. Our findings raise the possibility that patients with preserved systole and impaired diastole could have aberrations in cAMP signaling from the Golgi caused by a reduced receptor pool at the Golgi, impaired expression or reduced plasma membrane localization of OCT3 or reduced activity of downstream PKA effectors such as PLB. Establishing the physiological significance of GPCR/cAMP signaling from subcellular compartments in healthy cardiomyocytes is the first step in unraveling how this signaling specificity goes awry to cause cardiac disease.

## Methods

### Reagents and antibodies

Human insulin, human transferrin and sodium selenite (ITS); urethane; 2,3-butanedione monoxime (BDM); Taurine; protease XIV; polybrene; forskolin (FSK); epinephrine; dobutamine; corticosterone and IBMX are from Sigma. Glutamax solution, penicillin and streptomycin, 4-(2-hydroxyethyl)-1-piperazineethanesulfonic acid (HEPES) buffer, Hanks' Balanced Salt Solution (HBSS) buffer, M199 medium, ultrapure H_2_O, DMEM, mouse laminin and Halt protease and phosphatase inhibitor cocktail are from Thermo Fisher Scientific. FBS and Nu-Serum IV are from Corning. Glucose, sodium chloride, potassium chloride, sodium phosphate monobasic monohydrate, magnesium chloride hexahydrate, Tris–base, K-pipes, HEPES, ethylenediaminetetraacetic acid (EDTA), dithiothreitol (DTT), dimethyl sulfoxide (DMSO) and Tween-20 are from Fisher Bioreagents. Calcium chloride, Trolox and tricaine are from Acros Organics. ICI-118551 is from Tocris Bioscience. Doxycycline is from Takara. Heparin solution is from Fresenius Kabi. Collagenase II is from Worthington. Bovine serum albumin (BSA) and dry milk powder are from Research Products International. Ethylen glyco tetraacetic acid (EGTA) is from Alfa Aesar. Triton X-100 is from Bio-Rad. Proteinase K is from Roche. Rabbit anti-phospho PLB (Ser16/Thr17) antibody (8496) and rabbit anti-phospho TnI (Ser23/Ser24) antibody (4004) are from Cell Signaling. Rabbit anti-phospho ryanodine receptor 2 (Ser2808) antibody (PA5-104444) is from Thermo Fisher Scientific. Rabbit anti-calsequestrin 2 (CSQ2) antibody (18422-1-AP) and rabbit anti-SLC22A1 antibody (24617-1-AP) are from Proteintech. Rabbit anti-SLC22A3 antibody (ab183071) and rabbit anti-β1AR (ab3442) are from Abcam. Goat anti-β1AR antibody (EB07133) is from Everest Biotech. Rabbit anti-SNAP tag antibody (P9310S) is from New England Biolabs. Mouse anti-GM130 (610822) is from BD Biosciences. Sheep anti-TGN38 (AHP499G) is from Bio-Rad. Mouse anti-α-actinin (A7811) is from Sigma. Donkey anti-mouse IgG (H + L) highly cross-adsorbed secondary antibody (Alexa Fluor 647, A31571), donkey anti-rabbit IgG (H + L) highly cross-adsorbed secondary antibody (Alexa Fluor 647, A31573), donkey anti-sheep IgG (H + L) cross-adsorbed secondary antibody (Alexa Fluor 488, A11015), donkey anti-rabbit IgG (H + L) highly cross-adsorbed secondary antibody (Alexa Fluor 488, A21206), donkey anti-mouse IgG (H + L) highly cross-adsorbed secondary antibody (Alexa Fluor 488, A21202) and donkey anti-sheep IgG (H + L) cross-adsorbed secondary antibody (Alexa Fluor 555, A21436) were purchased from Thermo Fisher Scientific. Amersham ECL donkey anti-rabbit IgG (NA934V), horseradish peroxidase (HRP)-linked whole antibodies were purchased from GE Healthcare Life Sciences.

### Plasmid construction

pLVXTetOne lentiviral vector (a gift from Jura Lab) was used for doxycycline-induced protein expression in mouse neonatal cardiomyocytes. To generate pLVXTetOne signal peptide (SS)-SNAP-TGNP-bPAC plasmids, DNA fragments of SS-SNAP were amplified from pcDNA3_SS-SNAP-ADRB2 (a gift from von Zastrow Lab). The fragments of bPAC and TGNP were amplified from cytoplasmic-bPAC (a gift from Reiter Lab) and pmApple-TGNP-N-10 (Addgene plasmid, 54954), respectively. To generate pLVXTetOne-dnPrkar1a-msfGFP-CAAX, the DNA fragments of dnPrkar1a, msfGFP and CAAX were cloned from pCS2+ dnPKA-GFP (a gift from R. Moon; Addgene, 16716), msfGFP containing plasmid (a gift from Giacomini Lab) and pHR-SFFVp-CIB-GFP-CAAX (a gift from Weiner Lab). To generate pLVXTetOne_SS-SNAP-β1AR, SS-SNAP was cloned as previous description and β1AR was cloned from pcDNA3_SS-FLAG-β1AR (a gift from von Zastrow Lab). The cloned DNA fragments were inserted into the pLVXTetOne lentiviral vector (a gift from Jura Lab). To generate the pminiTol2 cmlc2: GalT-bPAC, the bPAC was amplified from cytosolic bPAC (a gift from Reiter Lab), GalT and mApple were amplified from the FKBP-GalT-mApple plasmid and inserted into the pminiTol2 cmlc2 vector (a gift from von Zastrow Lab). The DNA fragments were amplified by Pfu Ultra II Hotstart PCR master mix (Agilent Technologies) and ligated with each respective vector by NEBbuilder HiFi DNA assembly master mix (New England BioLabs).

### Cell culture and lentivirus production

HEK293, HeLa and HEK293T cells are cultured in DMEM (11965092) containing 10% FBS. Lentiviral vector was cotransfected with pSPAX2 and pMD2.G plasmids (gifts from Julius Lab) to HEK293T by TransIT-Lenti transfection reagent (Mirus Bio). The lentivirus was produced in DMEM containing 10% FBS and 1% BSA and then concentrated by the Lenti-X concentrator (Takara Bio).

### Animals

CD1, WT C57BL/6, *Slc22a3*-null C57BL/6 and *Adrb1*^*tm1Bkk*^ *Adrb2*^*tm1Bkk*^/J mice (003810) were housed in the facilities controlled by standardized environmental parameters, including 12 h light/12 h dark cycle in 7 d per week, humidity 30–70%, temperature 20–26 °C and access to water and foods ad libitum. All animal experiments were approved by the Institutional of Animal Care and Use Committee of the University of California, San Francisco. Genotyping of WT and *Slc22a3* null alleles were performed, as previously described^71^. The primer sets for the genotyping are as follows: WT allele (F: 5′-gttctggcctaggcagtgcctctaat-3′ and R: 5′-gtgctaatgacaacacatggagatg-3′; 300 bp) and Slc22a3-null allele (F: 5′-ggtactattcctcttgccaatcc-3′ and R: 5′-gtgctaatgacaacacatggagatg-3′; 500 bp). Genotyping of *Adrb1*^*tm1Bkk*^ *Adrb2*^*tm1Bkk*^/J mice was performed based on the protocols and primer information on the Jackson Laboratory website. Genotyping was performed using the GoTaq Green master mix (Promega).

Zebrafish were reared and handled in compliance with standard laboratory practices and institutional animal care and use committee (IACUC) protocols. Embryos were maintained in egg water at 28 °C in the dark for 5 d and then raised in a 14 h light/10 h dark cycle. Experimental embryos were assayed within 72 hpf at which time sex cannot be easily identified. However, sex is unlikely to affect the signaling pathways and physiological outputs in this study. GalT-bPAC fish were generated through the Tol2 transposon transgenesis of an established zebrafish line, Tg(Flk:Ras-cherry)^s896^ (refs. ^72,73^). Embryos were co-injected (PV pneumatic pico pump) at the one-cell stage with the pminiTol2 cmlc2: GalT-bPAC (4.5 pg) linear plasmid and capped transposase RNA (6.3 pg). Embryos positive for cherry fluorescence were sorted and genotyped. Genotyping to identify bPAC and Drer_Chr1 (DNA extraction control) was performed using the GoTaq Green master mix (Promega). DNA samples for genotyping were extracted (lysis buffer: 10 mM Tris (pH 8) 2 mM EDTA, 0.2% Triton X-100 and 200 μg ml^−1^ Proteinase K) from adult caudal fin clipping. The primer pairs used for genotyping are as follows: bPAC (F: 5′-gtcaaccggtacttcagcatct-3′ and R: 5′-tcgtagtacttctgggcctcat-3′; 473 bp), GalT (F: 5′-tgatccggcagaccctggaa-3′; and R: 5′-gccctcgatctcgaactcgt-3′: 470 bp), mApple (F: 5′-ggctccaaggtctacattaagcac-3′; and R: 5′-tgtagtcctcgttgtgggac-3′: 424 bp), Drer_ch1 (F: 5′-tatacgcggccataagtactga-3′ and R: 5′-gttcatttggggctttgggtat-3′; 218 bp). To determine mating pairs of GalT-bPAC fish, cAMP measurements were performed. Embryos at 72 hpf obtained from each pair were incubated with IBMX (100 μM, 90 min, 28 °C). Anaesthetization by incubation of tricaine (0.04% wt/vol) was confirmed by a reflex test of the tail. Embryos were then exposed to 4.2 μW cm^−^^2^ blue light to stimulate GalT-bPAC or maintained in the dark for 5 min. Zebrafish were lysed in 120 μl of 0.1 M HCl, and then cAMP was detected by a direct determination ELISA assay. Mating pairs that produced embryos that robustly generated cAMP in response to blue light were subsequently used in imaging experiments.

### Primary culture of cardiomyocytes

The processes for neonatal cardiomyocytes isolation are modified from the previous research^74^. Briefly, hearts collected from P1–2 neonatal CD1 pups were torn into small pieces in the ice-cold HBSS containing 20 mM HEPES. Heart pieces mixed with 225 IU ml^−1^ collagenase II were incubated on the tube rotator at 37 °C for 5 min. After 10-time pipetting, the released cells in the buffer were collected by centrifuge at 500*g* for 5 min. The undigested heart tissues were digested again, as described above, until the undigested tissue became white and the size do not decrease. The cells from each digestion were pooled together and resuspended in the neonatal cardiomyocyte culture media, which is DMEM (11995065) containing 10% FBS, 10% Nu-Serum IV, 10 mM HEPES, 10 mM Glutamax, penicillin and streptomycin and ITS. The released cells pass through a 40 µm strainer plated on the regular dish to remove the most of fibroblasts at 37 °C for 2 h. The suspended cells were collected and plated on the mouse laminin-coated dish. For the virus transduction, lentivirus was mixed with the culture media with polybrene (8 µg ml^−1^). The lentivirus was removed after 1-d transduction. The transduced neonatal cardiomyocytes were further treated with doxycycline for 3 d.

Adult cardiomyocytes were isolated from 2- to 3-month-old C57BL/6 WT and *Slc22a3* knockout mice using the Langedorff-free method^75^. The heparin solution was intraperitoneally injected into the mouse (5 U g^−1^). After 10 min, urethane, dissolved in 0.9% NaCl, was also intraperitoneally injected into the mouse (2 mg g^−1^). When the mouse was fully killed, the mouse’s heart was exposed and the inferior vena cava was cut to release the blood. After the injection of EDTA buffer (130 mM NaCl, 5 mM KCl, 0.5 mM NaH_2_PO_4_-H_2_O, 10 mM HEPES, 10 mM glucose, 10 mM BDM, 10 mM Taurine, in ultrapure H_2_O) into the right ventricle, the aorta was clamped. The clamped heart was moved to the EDTA buffer-containing dish and then the EDTA buffer was injected into the left ventricle. Then, the clamped heart was moved to the perfusion buffer (130 mM NaCl, 5 mM KCl, 0.5 mM NaH_2_PO_4_–H_2_O, 10 mM HEPES, 10 mM glucose, 10 mM BDM, 10 mM Taurine, 1 mM MgCl_2_–6H_2_O, in ultrapure H_2_O) containing dish and the perfusion buffer was injected into the left ventricle. The clamped heart was further moved to the digestion buffer (perfusion buffer with 0.5 mg ml^−1^ collagenase II and 0.05 mg ml^−1^ protease XIV) containing dish, and then the digestion buffer was injected in to left ventricle. After digestion, the heart was torn into small pieces and gently triturated to dissociate the cardiomyocytes. The digestion processes were stopped by adding stop buffer (perfusion buffer with 5% FBS), and the suspended cardiomyocytes were passed through the 100 µm strainer. The cardiomyocytes were enriched by gravity sedimentation and reintroduced calcium gradually. The cardiomyocytes were resuspended by plating media (M199 media with 5% FBS, 10 mM BDM, penicillin and streptomycin) and plated on the mouse laminin-coated wells at 37 °C for 1 h. After washing out the unattached cells by culture media (M199 media with 0.1% BSA, 10 mM BDM, penicillin and streptomycin and ITS), the cardiomyocytes were cultured in culture media for further use.

### Blue light stimulation for activating bPAC protein in cardiomyocytes

After 1-d transduction, neonatal cardiomyocytes were treated with 100 ng ml^−1^ doxycycline for 3 d and then treated with 100 μM Trolox for 4 h. bPAC-expressing neonatal cardiomyocytes were put under the blue LED board in the incubator. After blue light stimulation for indicated interval, the neonatal cardiomyocytes were washed with ice-cold PBS once and lysed. To measure the blue light intensity, we used a Digital Handheld Optical Power and Energy Meter Console (PM100D, Thorlabs) with a Slim Photodiode Power Sensor probe (S130C, Thorlabs). The light intensities were calculated from the power measured (*W*) and the probe detection surface of 0.7855 cm^2^.

### Fixed-cell confocal imaging

For the staining of Golgi-bPAC and PM-dnPKA in the neonatal and adult cardiomyocytes or HeLa cells, cells were washed with PBS once and fixed with 3.7% formaldehyde in PEM buffer (80 mM K-PIPES (pH 6.8), 1 mM MgCl_2_ and 1 mM EGTA) for 20 min at room temperature. For the staining of pRyR2 Ser2808, pTnI Ser23/Ser24, pPLB Ser16/Thr17, PLB and β1AR, cells were prepermeabilized with 0.05% saponin diluted in PEM buffer on ice for 5 min before fixation. Fixation was performed using 3% paraformaldehyde diluted in PBS for 10 min at room temperature and further quenched by 50 mM NH_4_Cl diluted in PBS for 10 min. Fixed cells were incubated with the primary antibody at room temperature for 1 h or at 4 °C for O/N in TBS containing 0.1% Triton. After the incubation with the secondary antibody at room temperature for 30 min, cells were mounted using anti-fade mounting medium with DAPI (Vector Laboratories). The images were taken by Nikon spinning disk confocal microscope using NIS Elements (v5.02).

### Lysate preparation, SDS–PAGE and western blot analysis

After the treatments, the cardiomyocytes from neonatal and adult mice were collected and lysed by radio-immunoprecipitation assay (RIPA) buffer containing inhibitors of proteases and phosphatases at 4 °C for 30 min on the tube rotator. Supernatants were collected after centrifuging at 4 °C for 10 min, and the protein amounts were determined by BCA assay (Sigma). The proteins were denatured by boiling for 10 min in the DTT-containing sample buffer and separated by 4–20% Mini-PROTEIN TGX gels (Bio-Rad) and then transferred to the 0.2 µm PVDF membrane (Bio-Rad). The polyvinylidene difluoride (PVDF) membrane was further blocked by TBST (TBS buffer with 0.1% Tween-20) containing 3% milk at room temperature for 1 h and then incubated with the primary antibody in TBST containing 5% BSA at 4 °C for O/N. The PVDF membrane was washed by TBST three times and then incubated with a secondary antibody diluted in TBST containing 3% milk at room temperature for 1 h. The unbonded secondary antibodies were removed by three times washing using TBST. The protein signals were visualized by ECL substrate (Thermo Fisher Scientific). To evaluate the relative band intensities, we first scanned our films (300 ppi resolution) using an office scanner and convert them to an 8-bit format. We then inverted these images and subtracted the background using ImageJ software Iv.1.53f). The bands were selected using the rectangular selection tool on ImageJ. The relative band intensities were measured, and each peak was separated by straight-line selection tool. The area of each peak was measured by the Wand tool. Semi-quantified phosphoprotein bands were then normalized to the total lysate bands (CSQ2), and data were presented as percentages of the maximum value that we measured on each western blot.

### Intracellular calcium imaging

Local increase in intracellular Ca^2+^ concentration was recorded using line-scanning confocal of isolated adult cardiomyocytes. Adult cardiomyocytes plated in Tyrode’s solution (140 mM NaCl, 4 mM KCl, 1.1 mM MgCl_2_, 10 mM HEPES, 10 mM glucose, 1.8 mM CaCl_2_; pH = 7.4, with NaOH) and were loaded with 5 μM Ca^2+^ dye Fluo-4 AM ester (Invitrogen Life Technologies) for 20 min at room temperature. Images were obtained using a Zeiss LSM 780-FLIM confocal microscope (×40 water objective). Fluo-4 AM was excited at 488 nm, and emitted fluorescence was collected at 505 nm. The confocal pinhole is set to render a 1.4 μm section. The gain was set at 800. To obtain intracellular Ca^2+^ transients, adult cardiomyocytes loaded with Fluo-4 AM were electrically excited at 1 Hz for 15 s by filed stimulation using Ionoptix MyoPacel filed stimulator. A line scan image (256 pixels) was acquired at a rate of 0.473 ms per line along the longitudinal axis of the cells. The fluorescent values (*F*) were normalized to the basal fluorescence (*F*0) to obtain the fluorescence ratio *F*/*F*0 before and after 1 μM epinephrine stimulation. The Ca^2+^ decay tau was calculated by fitting the decay trace of 80% of the maximum response, using Graphpad Prism v9.3.1.

### Zebrafish imaging and analysis

Dechorionated embryos at 72 hpf were pretreated with a DMSO control (0.1% vol/vol) or IBMX (100 μM, 90 min) and were anesthetized with tricaine (0.04% wt/vol). Embryos were then mounted onto glass bottom imaging dishes (35 mm, MatTek) with low melting agarose (1% wt/vol) and maintained in tricaine for the duration of the experiment. All image series were acquired at 61 fps for 500 frames (*λ*_ex_ = 561 nm and *λ*_em_ = 640 nm) with a Plan Apo 40X air objective (Nikon) on a spinning disk confocal (Nikon Eclipse Ti). Baseline images were obtained, and GalT-bPAC was stimulated by exposing embryos to 4.2 μW cm^−^^2^ of blue light for time points up to 5 min. The data were analyzed using OriginPro software (v9.8.0.200) and FIJI (ImageJ v1.53f) by measuring the fluorescence intensity of a ventricular region of interest (ROI) and determining the time between peak contraction and peak relaxation. The time between fluorescence minima to maxima is the time of relaxation, and the subsequent fluorescence maxima to minima portion of the graph is measured as the time of contraction. Heart rates were calculated by measuring the distance between the two consecutive highest points of the peaks of each cycle. Graphs were generated using Graphpad Prism v9.3.1. All data are expressed as Δ time (ms) relative to the baseline, as a mean ± s.e.m.

### Mouse cardiac PV loop acquisition and analysis

To assess ventricular systolic and diastolic function, we conducted PV loop experiments using a conductance catheter (Millar Instruments) in mice, as described in a previous study^76^. Briefly, pressure and conductance calibrations were performed. Mice were initially anesthetized by inhalation of isoflurane (1.5% mixed with 100% oxygen). An endotracheal tube was placed and connected to the ventilator, and ventilator settings were based on animal weight^77^. Mice were placed on a heating pad, and body temperatures were maintained at 37 °C. Subsequently, analgesia was administered by subcutaneous injection of buprenorphine (0.05 mg kg^−1^). Proper anesthetization was confirmed by a reflex test of the tail. A total of 1 mg kg^−1^ pancuronium (Sigma Life Science) was injected intraperitoneally to prevent respiratory artifacts during recordings^78^. The aortic arch and inferior vena cava were exposed with a 6-0 silk ligature placed underneath separately. The right jugular vein was cannulated for subsequent fluid and medication infusion. A thoracotomy was performed, and the pericardium was bluntly dissected to expose the left ventricular apex. A 25-gauge needle was used to make a stab incision of the apex, followed by the insertion of a 1.4-F pressure-conductance catheter (PVR-1035, Millar Instruments) through the incision. The intraventricular catheter position was optimized until rectangular-shaped loops were obtained (LabChart 8.5 Pro). Then, 200 µl 0.9% saline was perfused slowly to replace body fluid loss. After steady-state conditions were reached in 10 min, baseline PV loops were recorded with three cycles of inferior vena cava and transverse aorta occlusion in sequence. Then epinephrine (10 μg kg^−1^) or dobutamine (18.4 μg kg^−1^) was injected, and the above steps were repeated. To estimate *G*_p_, 10 μl hypertonic saline (15% NaCl) was rapidly injected at the end of the experiment. After 5 min, the blood was collected from the right ventricle for a cuvette calibration to transform conductance to volume. Cardiac parameters were obtained by offline data analysis on LabChart software (8.5). Shifts in the loops between basal and stimulated conditions provide a comprehensive analysis of cardiac function and can be used to assess the heart’s performance by quantitatively measuring hemodynamic parameters. Multiple cardiac indicators, including the end-systolic pressure–volume relationship, end-diastolic pressure–volume relations, stroke volume, cardiac output and ejection fraction, can be derived from PV loops.

### cAMP determination

Two modes of cAMP determination were performed. A direct cAMP ELISA kit (Enzo) was used in endpoint experiments, and the pGloSensor (-20F) luminescence assay (Promega) was used for kinetic cAMP measurements. For time-dependent cAMP production assays in HEK293 cells, cells were transiently transfected with GalT-bPAC and the pGloSensor-20F plasmid. Twenty-four hours after transfection, cells were incubated with Glosensor cAMP reagent for 1 h at 37 °C. For GalT experiments, cells were exposed to 4.2 μW cm^−^^2^ blue light for up to 300 s. Luminescence measurements were acquired at 2 min intervals. Three baseline measurements were acquired, after which cells were stimulated and measured for at least 20 min. The specified cells were treated with the PDE inhibitor IBMX (100 μM) and positive control, FSK (20 μM). For primary cell and zebrafish cAMP determination, ELISA assays were performed to determine cAMP. To measure epinephrine-induced cAMP production in mouse neonatal cardiomyocytes, cells were treated with a range of epinephrine (2 min, 37 °C). Cells were immediately lysed in 175 μl of 0.1 M HCL lysis buffer for the direct cAMP determination by ELISA, which was performed as directed in the kit. To measure cAMP concentration in zebrafish, fifteen 72 hpf zebrafish per condition were lysed in 175 μl of 0.1 M HCL lysis buffer. In total, 100 μM FSK-treated zebrafish were used as a positive control. SoftmaxPro (v7.03) was used to collect data from the plate reader.

### Statistics

All cardiac parameters from PV looping analysis are presented as mean ± s.d., and SigmaStat 3.5 was used for comparison. A paired *t-*test was used to compare data in the same group before and after chemical infusion, while other data between groups were compared with one-way ANOVA. The significant difference between groups in dnPKA-related experiments was determined using two-way ANOVA. A post hoc Student–Newman–Keuls test was further conducted to compare differences between the two groups. A *P* < 0.05 was considered significant.

### Reporting summary

Further information on research design is available in the Nature Portfolio Reporting Summary linked to this article.

## Online content

Any methods, additional references, Nature Portfolio reporting summaries, source data, extended data, supplementary information, acknowledgements, peer review information; details of author contributions and competing interests; and statements of data and code availability are available at 10.1038/s41589-023-01381-8.

### Supplementary information

Supplementary Information Supplementary Table 1: Heart rate, maximal and minimal rate of left ventricle pressure change (dp/dt max and dp/dt min), tau, ejection fraction, and cardiac output of both wild-type and OCT3 knockout mice were measured upon injection of epinephrine and dobutamine.

Reporting Summary

### Source data

Source Data Fig. 1 Unprocessed western blots and image files/statistical source data.

Source Data Fig. 2 Statistical source data.

Source Data Fig. 3 Unprocessed western blots/statistical source data.

Source Data Fig. 4 Unprocessed western blots and image files/statistical source data.

Source Data Fig. 5 Unprocessed western blots and image files/statistical source data.

Source Data Fig. 6 Statistical source data.

Source Data Extended Data Fig 1 Unprocessed western blots and image files/statistical source data.

Source Data Extended Data Fig. 2 Statistical source data.

Source Data Extended Data Fig. 3 Unprocessed image files.

Source Data Extended Data Fig. 4 Unprocessed image files/statistical source data.

Source Data Extended Data Fig. 5 Unprocessed western blots and image files/statistical source data.

Source Data Extended Data Fig. 6 Unprocessed western blots/statistical source data.

Source Data Extended Data Fig. 7 Unprocessed image files/statistical source data.

Source Data Extended Data Fig. 8 Unprocessed image files.

## Data Availability

All data generated and analyzed during this study are included in this manuscript as figures, extended data figures, tables and source data. Transgenic animals are readily available upon request. Source data are provided with this paper.
