# Supplementary material for: Cardiac contraction and relaxation are regulated by distinct subcellular cAMP pools
Source: Nat Chem Biol. Author manuscript; Available in PMC 2024 Feb 16. (PMC10746541; doi:10.1038/s41589-023-01381-8)
Supplement: Supplementary table [file NIHMS1920068-supplement-Supplementary_table.pdf]

nature chemical biology

Article https://doi.org/10.1038/s41589-023-01381-8

Cardiac contraction and relaxation are regulated by distinct subcellular cAMP pools

In the format provided by the authors and unedited

	CO uL/min (Bl)	CO uL/min (Epi)	EF % (BL)	EF % (Epi)	HR bpm (BL)	HR bpm (Epi)
WT mice baseline vs Epi	6068	7822	65.66	92.07	543.5	609.2
	2987	4707	37.52	63.47	617.4	664.5
	6915	6579	48.43	50	556.9	611
	5363	6392	50.4	53.48	593.5	645.9
	6470	7189	39.54	50.45	586	692.1
	6529	9464	44.56	60.54	587.7	669.7
	5819	9847	44.19	77.59	651.5	718.6
	4400	5451	56.71	87.55	516.4	604.8
	4686	8565	28.61	54.32	526.3	668.2
	9711	9166	68.49	78.21	642.4	651.9

	CO uL/min (Bl)	CO uL/min (Epi)	EF % (BL)	EF % (Epi)	HR bpm (BL)	HR bpm (Epi)
WT mice baseline vs Epi	6068	7822	65.66	92.07	543.5	609.2
	2987	4707	37.52	63.47	617.4	664.5
	6915	6579	48.43	50	556.9	611
	5363	6392	50.4	53.48	593.5	645.9
	6470	7189	39.54	50.45	586	692.1
	6529	9464	44.56	60.54	587.7	669.7
	5819	9847	44.19	77.59	651.5	718.6
	4400	5451	56.71	87.55	516.4	604.8
	4686	8565	28.61	54.32	526.3	668.2
	9711	9166	68.49	78.21	642.4	651.9

	CO uL/min (Bl)	CO uL/min (Dob)	EF % (BL)	EF % (Dob)	HR bpm (BL)	HR bpm (Dob)
WT mice baseline vs Dob	4069	4958	65.96	74.94	594.8	603.6
	4444	6600	69.87	91.21	552	602.3
	3951	5194	51.94	67.08	692.9	692.7
	4563	6567	60.18	44.03	567.1	602
	4076	6370	56.19	77.42	600	612.2
	4315	5548	44.33	62.37	553	598.8
	4253	4367	64.4611	66.32937	685.7	665.9

	CO uL/min (Bl)	CO uL/min (Dob)	EF % (BL)	EF % (Dob)	HR bpm (BL)	HR bpm (Dob)
OCT3 KO mice baseline vs Dob	5674	13400	74.55	89.36	636.4	665.6
	5372	6841	70.82	88.76	682	705
	5337	6252	60.06	67.15	647.2	673.3
	7552	11300	73.94	82.05	604.8	623.7
	3223	7748	35.88	60.59	500.7	619.4
	6786	7432	48.2	68.53	653.1	635
	2992	4761	38.16	52.92	582	588.8
	3843	4441	46.1	51.06	525.9	550
	4844	6647	58.8	72.45	580.9	598.8
	7309	9897	58.47	68.47	585.4	609.8
