# Supplementary material for: Cardiac contraction and relaxation are regulated by distinct subcellular cAMP pools
Source: Nat Chem Biol. 2023 Jul 20;20(1):62–73. doi: 10.1038/s41589-023-01381-8 (PMC10746541; doi:10.1038/s41589-023-01381-8)
Supplement: Supplementary file 1 [file 41589_2023_1381_MOESM2_ESM.pdf]

nature portfolio

Corresponding author(s): Roshanak Irannejad

Last updated by author(s): 2023/05/17

Reporting Summary

Nature Portfolio wishes to improve the reproducibility of the work that we publish. This form provides structure for consistency and transparency in reporting. For further information on Nature Portfolio policies, see our Editorial Policies and the Editorial Policy Checklist.

Statistics

For all statistical analyses, confirm that the following items are present in the figure legend, table legend, main text, or Methods section.

n/a	Confirmed	
☐	☒	The exact sample size (*n*) for each experimental group/condition, given as a discrete number and unit of measurement
☐	☒	A statement on whether measurements were taken from distinct samples or whether the same sample was measured repeatedly
☐	☒	The statistical test(s) used AND whether they are one- or two-sided<br>*Only common tests should be described solely by name; describe more complex techniques in the Methods section.*
☒	☐	A description of all covariates tested
☐	☒	A description of any assumptions or corrections, such as tests of normality and adjustment for multiple comparisons
☐	☒	A full description of the statistical parameters including central tendency (e.g. means) or other basic estimates (e.g. regression coefficient) AND variation (e.g. standard deviation) or associated estimates of uncertainty (e.g. confidence intervals)
☐	☒	For null hypothesis testing, the test statistic (e.g. *F*, *t*, *r*) with confidence intervals, effect sizes, degrees of freedom and *P* value noted<br>*Give P values as exact values whenever suitable.*
☒	☐	For Bayesian analysis, information on the choice of priors and Markov chain Monte Carlo settings
☒	☐	For hierarchical and complex designs, identification of the appropriate level for tests and full reporting of outcomes
☒	☐	Estimates of effect sizes (e.g. Cohen's *d*, Pearson's *r*), indicating how they were calculated

Our web collection on statistics for biologists contains articles on many of the points above.

Software and code

Policy information about availability of computer code

Data collection	NIS Elements (vS.02) was used to collect microscopy images. SoftmaxPro (v7.0.3) was used to collect data for all plate-based assays.
Data analysis	Graphpad Prism (v9.3.1) was used for statistical analyses. Fiji (ImageJ v.l.53f) and the wand tool included in the ImageJ v.l.53f software was used for imaging analyses. OriginPro Software (v9.8.0.200) and peak analyzer module (included with OriginPro software(v9.8.0.200)) was used for zebrafish analyses. abChart 8.5 Pro was used for the PV loop analysis.

For manuscripts utilizing custom algorithms or software that are central to the research but not yet described in published literature, software must be made available to editors and reviewers. We strongly encourage code deposition in a community repository (e.g. GitHub). See the Nature Portfolio guidelines for submitting code & software for further information.

Data

Policy information about availability of data

All manuscripts must include a data availability statement. This statement should provide the following information, where applicable:

- Accession codes, unique identifiers, or web links for publicly available datasets
- A description of any restrictions on data availability
- For clinical datasets or third party data, please ensure that the statement adheres to our policy

All data generated or analyzed during this study are included in this article and its supplementary information files.

Research involving human participants, their data, or biological material

Policy information about studies with human participants or human data. See also policy information about sex, gender (identity/presentation), and sexual orientation and race, ethnicity and racism.

Reporting on sex and gender	*Use the terms sex (biological attribute) and gender (shaped by social and cultural circumstances) carefully in order to avoid confusing both terms. Indicate if findings apply to only one sex or gender; describe whether sex and gender were considered in study design; whether sex and/or gender was determined based on self-reporting or assigned and methods used. Provide in the source data disaggregated sex and gender data, where this information has been collected, and if consent has been obtained for sharing of individual-level data; provide overall numbers in this Reporting Summary. Please state if this information has not been collected. Report sex- and gender-based analyses where performed, justify reasons for lack of sex- and gender-based analysis.*
Reporting on race, ethnicity, or other socially relevant groupings	*Please specify the socially constructed or socially relevant categorization variable(s) used in your manuscript and explain why they were used. Please note that such variables should not be used as proxies for other socially constructed/relevant variables (for example, race or ethnicity should not be used as a proxy for socioeconomic status). Provide clear definitions of the relevant terms used, how they were provided (by the participants/respondents, the researchers, or third parties), and the method(s) used to classify people into the different categories (e.g. self-report, census or administrative data, social media data, etc.) Please provide details about how you controlled for confounding variables in your analyses.*
Population characteristics	*Describe the covariate-relevant population characteristics of the human research participants (e.g. age, genotypic information, past and current diagnosis and treatment categories). If you filled out the behavioural & social sciences study design questions and have nothing to add here, write "See above."*
Recruitment	*Describe how participants were recruited. Outline any potential self-selection bias or other biases that may be present and how these are likely to impact results.*
Ethics oversight	*Identify the organization(s) that approved the study protocol.*

Note that full information on the approval of the study protocol must also be provided in the manuscript.

Field-specific reporting

Please select the one below that is the best fit for your research. If you are not sure, read the appropriate sections before making your selection.

☒ Life sciences ☐ Behavioural & social sciences ☐ Ecological, evolutionary & environmental sciences

For a reference copy of the document with all sections, see nature.com/documents/nr-reporting-summary-flat.pdf

Life sciences study design

All studies must disclose on these points even when the disclosure is negative.

Sample size	All sample sized were determined base on previous experience and published literature (PMID: 33932338, PMID: 28553949, PMID: 36626303). 38 zebrafish, 10-11 mice and 15 cells were used for each condition in the respective experiments. All sample sizes and statistical analysis are specified in figure, figure legends and method sections (pages 1-0-20). Indivitual dots represent independent biological replicates or individual animals.
Data exclusions	No data were excluded.
Replication	Biological replicates are defined as distinct sets of western blots on separate experimental days, or distinct cardiomyocytes or other cell types for cell culture experiments. All in vivo zebrafish microscopy measurements were repeated at least six independent imaging sessions. zebrfish genotyping were performed after each session (at least six independent times). All in vivo mouse analyses were repeated three independent sessions. All cardiomyocyte cultures for western blots were performed at least six independent sessions. Mice were geneotyped after each session (at least six independent time). All cardiomyocyte cultures for fixed cell imaging were performed at least three independent sessions.
Randomization	Randomization was not necessary for these experiments. Experiemtns were performed based on genotype (for mice and zebrafish) and treatment (for cardiomyocytes).
Blinding	Investigators were blinded for fixed imaging of cardiomyocytes derived from wild-type and beta1/2AR knockout mice. For all other experiments performed with isolated cardiomyocytes, zebrafish or mice, animals were genotyped beforehand and thus blinding was not required.

Reporting for specific materials, systems and methods

We require information from authors about some types of materials, experimental systems and methods used in many studies. Here, indicate whether each material, system or method listed is relevant to your study. If you are not sure if a list item applies to your research, read the appropriate section before selecting a response.

Materials & experimental systems

n/a	Involved in the study	
☐	☒	Antibodies
☐	☒	Eukaryotic cell lines
☒	☐	Palaeontology and archaeology
☐	☒	Animals and other organisms
☒	☐	Clinical data
☒	☐	Dual use research of concern
☒	☐	Plants

Methods

n/a	Involved in the study	
☒	☐	ChIP-seq
☒	☐	Flow cytometry
☒	☐	MRI-based neuroimaging

Antibodies

Antibodies used

Rabbit anti-phospho phospholamban (Ser16/Thr17) antibody, Cell Signaling, #8496
Rabbit anti-phospho troponin I (Ser23/24) antibody, Cell Signaling, #4004
Rabbit anti-phospho ryanodine receptor 2 (Ser2808) antibody, Thermo Fisher Scientific, #PA5-104444
Rabbit anti-Calsequestrin 2 antibody, Proteintech, #18422-1-AP
Rabbit anti-SLC22A1 antibody, Proteintech, #24617-1-AP
Rabbit anti-SLC22A3 antibody, Abcam, #ab183071
Rabbit anti-β1AR, Abcam, #ab3442
Goat anti-β1AR antibody, Everest Biotech, #EB07133
Rabbit anti-SNAP tag antibody, New England Biolabs, #P9310S
Mouse anti-GM130, BD Biosciences, #610822
Sheep anti-TGN38, Bio-Rad, #AHP499G
Mouse anti-α-actinin, Sigma, #A7811
Amersham ECL Donkey anti-Rabbit IgG, HRP-linked whole antibody, GE Healthcare Life Sciences, #NA934V
Donkey anti-Mouse IgG (H+L) Highly Cross-Adsorbed Secondary Antibody, Alexa Fluor™ 647, Thermo Fisher Scientific, #A31571
Donkey anti-Rabbit IgG (H+L) Highly Cross-Adsorbed Secondary Antibody, Alexa Fluor™ 647, Thermo Fisher Scientific, #A31573
Donkey anti-Sheep IgG (H+L) Cross-Adsorbed Secondary Antibody, Alexa Fluor™ 488, Thermo Fisher Scientific, #A11015
Donkey anti-Rabbit IgG (H+L) Highly Cross-Adsorbed Secondary Antibody, Alexa Fluor™ 488, Thermo Fisher Scientific, #A21206
Donkey anti-Mouse IgG (H+L) Highly Cross-Adsorbed Secondary Antibody, Alexa Fluor™ 488, Thermo Fisher Scientific, #A21202
Donkey anti-Sheep IgG (H+L) Cross-Adsorbed Secondary Antibody, Alexa Fluor™ 555, Thermo Fisher Scientific, #A21436

Validation

All antibodies used are standard commercial antibodies and validated by the respective manufacturer. Antibodies were validated based on expected molecular weight or using inhibitory peptide corresponding to the target protein. No custom antibody that required validation were generated.

Eukaryotic cell lines

Policy information about cell lines and Sex and Gender in Research

Cell line source(s)

Human embryonic kidney (HEK)293T cells: ATCC (CRL-11268)
Human embryonic kidney (HEK)293 cells: ATCC (CRL-1573)
HeLa Cells: ATCC (CCL-2)

Authentication

All cells were authenticated by ATCC, using morphology and SRT profiling.

Mycoplasma contamination

All cell lines tested negative for mycoplasma contamination.

Commonly misidentified lines
(See ICLAC register)

No commonly misidentified lines used.

Animals and other research organisms

Policy information about studies involving animals; ARRIVE guidelines recommended for reporting animal research, and Sex and Gender in Research

Laboratory animals

The Tg(Flk:Ras-cherry)s896 zebrafish line was used to generate the transgenic GalT-bPAC fish. Adult fish were housed at 28°C in a 14h light/10h dark cycle and were fed dry food twice daily (Zeigler Adult Zebrafish diet pellets) and live food once daily (brine shrimp). Embryos (both male and female) produced by GalT-bPAC and Tg(Flk:Ras-cherry)s896 fish were maintained in the dark and imaged within 72hpf.
CD-1 (Charles River, Cat#022), wild-type C57BL/6 (Charles River, Cat #027), Adrb1tm1Bkk Adrb2tm1Bkk/J mice (Jackson Laboratories, Cat #003810) and Slc22a3-null C57BL/6 were housed in the facilities controlled by standardized environmental parameters, including a 12h light/dark cycle 7 days per week, humidity 30-70%, temperature 20-26°C, and access to water and food ad libitum.
Neonatal mice (male and female) up to day 3 were used to prepare mouse primary neonatal cardiomyocytes.
Adult male mice up to 3 months were used to prepare mouse primary adult cardiomyocytes. Adult male mice of 3 months old were used for the PV-loop measurements.
All animal experiments were approved by the Institution of Animal Care and Use Committee of the University of California, San

	Francisco.
Wild animals	No wild animals were used.
Reporting on sex	For PV loop experiments and adult cardiomyocytes isolations, male mice of 3 months age were used.
Field-collected samples	No field-collected samples were used.
Ethics oversight	All animal experiments were approved by the Institutional of Animal Care and Use Committee of the University of California, San Francisco.

Note that full information on the approval of the study protocol must also be provided in the manuscript.
